# Whole-body scanning PCR; a highly sensitive method to study the biodistribution of mRNAs, noncoding RNAs and therapeutic oligonucleotides

**DOI:** 10.1093/nar/gkt515

**Published:** 2013-06-13

**Authors:** Julien A. Boos, David W. Kirk, Mari-Luz Piccolotto, Werner Zuercher, Sandro Gfeller, Philippe Neuner, Andre Dattler, William L. Wishart, Fabian Von Arx, Michael Beverly, Jesper Christensen, Karine Litherland, Esther van de Kerkhof, Pieter J. Swart, Thomas Faller, Armin Beyerbach, David Morrissey, Juerg Hunziker, Iwan Beuvink

**Affiliations:** ^1^Novartis Institutes for Biomedical Research (NIBR), Novartis Pharma AG, Basel, Basel-Stadt CH-4056, Switzerland and ^2^NIBR, Novartis Pharma AG, Cambridge, Massachusetts, MA 02139, USA

## Abstract

Efficient tissue-specific delivery is a crucial factor in the successful development of therapeutic oligonucleotides. Screening for novel delivery methods with unique tissue-homing properties requires a rapid, sensitive, flexible and unbiased technique able to visualize the *in vivo* biodistribution of these oligonucleotides. Here, we present whole body scanning PCR, a platform that relies on the local extraction of tissues from a mouse whole body section followed by the conversion of target-specific qPCR signals into an image. This platform was designed to be compatible with a novel RT-qPCR assay for the detection of siRNAs and with an assay suitable for the detection of heavily chemically modified oligonucleotides, which we termed Chemical-Ligation qPCR (CL-qPCR). In addition to this, the platform can also be used to investigate the global expression of endogenous mRNAs and non-coding RNAs. Incorporation of other detection systems, such as aptamers, could even further expand the use of this technology.

## INTRODUCTION

Since the first suggestion in 1978 that oligonucleotides might be used therapeutically, several classes of nucleic acid-based molecules and approaches have been described that could be used to modulate a large number of ‘undrugable’ targets in a wide variety of diseases. Their unique properties allow them to modulate various immunological responses (CpG oligonucleotides), inhibit or stimulate RNA transcription (triplex-forming oligonucleotides), correct splicing defects (antisense), inhibit the translation of proteins (siRNA, antisense, ribozymes), prevent protein interactions (aptamers, decoys) or restore the loss of function of various players in the signal transduction cascade of a cell (virus, plasmid). However, despite early promise, the development of nucleic acid-based therapeutics has proved to be a challenge owing to issues concerning stability, off-target effects and delivery efficiency (1,2). Improvements in oligonucleotide-design software and the development of various chemical modifications [e.g. phosphorothioate, locked nucleic acid, 2′-*o*-Methyl, 2′-*o*-(2-methoxyethyl), morpholino, 2′-fluoro] have greatly reduced the chance of off-target effects and increased the *in vivo* stability (3,4). Effective delivery of oligonucleotides to various tissues, on the other hand, remains a significant challenge in the development process. Most strategies used in the identification and characterization of novel oligonucleotide delivery systems rely on a primary screen performed *in vitro* using cell-based assays, which, in general, do not reflect the complex conditions encountered *in vivo*. Additional *in vivo* experiments are required to confirm and optimize the homing properties of the delivery vehicle. Although several invasive and non-invasive methods are reported to study the biodistribution of nucleic acids (5–7), most methods require the addition of modifications to either the oligonucleotide or the delivery vehicle, which could alter their biophysical properties and hence affect their true biodistribution (8,9). In addition to this, biodistribution patterns obtained by methods that rely on the direct labelling of oligonucleotides (e.g. radiolabelling, dyes) could lead to false-positive results owing to accumulation of metabolites in secondary tissues (10). To simplify the screening process of novel *in vivo*-targeted delivery strategies, we have developed a flexible platform, which we term whole body scanning polymerase chain reaction (WBS-PCR) that can be used to study the biodistribution of wild-type and chemically modified oligonucleotides as well as endogenous RNAs across whole body sections of mice. To demonstrate the broad utility of the technique, images of RNA distributions have been obtained using conventional mRNA Taqman RT-qPCR procedures, as well as with a novel reverse transcriptase quantitative polymerase chain reaction (RT-qPCR) assay to visualize the biodistribution of small RNAs such as miRNAs and siRNAs. We have developed the chemical-ligation qPCR (CL-qPCR), a novel template-mediated chemical ligation assay (11), for biodistribution studies involving heavily chemical-modified oligonucleotides such as 2′-*o*-(2-methoxyethyl)-modified antagomirs, which are typically not ameanable to enzyme-based detection methods. The WBS-PCR assay offers unique advantages in flexibility, sensitivity and specificity over other imaging technologies and could have broader applications than what is demonstrated here.

## MATERIALS AND METHODS

### Oligonucleotides

Synthetic RNA sequences and DNA PCR primers for miRNA, siRNA and antagomir detection were ordered from Microsynth AG (Switzerland) (Supplementary Table S5). TaqMan gene expression assays were ordered from Applied Biosystems (USA) (Supplementary Table S6).

### Design of miRNA and siRNA RT-qPCR primers

The RT-primer, which also serves as reverse primer during the PCR reaction, contains a generic sequence at the 5′ end and a Target-Recognition Sequence (TRS) at the 3′ end. The optimal TRS sequence in most assays consists of 8 nucleotides but can vary in length, ranging from 6 to 12 nucleotides, depending on the sequence of the target molecule. For example, if the 3′ end of the RT-primer ends with a palindromic motif (i.e. -ccgg, -aatt, -ggcc, -ttaa, -atat, -tata, -cgcg and -gcgc), the performance of the assay can be considerably improved by either removing or extending the TRS sequence by a single nucleotide so that the palindromic sequence is disrupted. The generic sequence is required to increase the melting temperature (Tm) of the oligonucleotide and to modulate the G/C-content, as the same primer will function as reverse primer in the subsequent PCR reaction.

The 5′-fluorophore-labelled forward primer contained a generic sequence at the 5′ end and a gene-specific region spanning at least 12 nucleotides at the 3′ end. The optimal length of the gene-specific region depends on the target sequence and the TRS length of the RT-primer. Excessive overlap (> three nucleotides) with the RT-primer and palindromic sequences at the 3′ end should be avoided. The generic sequence of the forward primer should be complementary to the quencher-labelled anti-primer sequence (5′-AAATCGAGGGAGGGAG-BHQ_2_-3′).

### Synthetic miRNA standard curves preparation

Synthetic miRNAs were serially diluted into Poly(A) (10 ng/μl diluted in RNAse-free water/GE Healthcare, #27-411-01) to generate a dilution series ranging from 10 ng/μl down to 1 zg/μl. A sample containing only 10 ng/μl Poly(A) in RNase-free water was included as No Template Control (NTC) and was used to determine the background level of RT-qPCR primers.

### siRNA duplex standard curve preparation

siRNA was serially diluted into 10 ng/μl total rat liver RNA to generate a dilution series ranging from 10 ng/μl down to 1 zg/μl. A sample containing only 10 ng/μl Poly(A) in RNase-free water was included as NTC and was used to determine the background level of RT-qPCR primers.

### AMO-miR-16 standard curves preparation

AMO-miR-16 was serially diluted in either Poly(A) (10 ng/μl diluted in RNAse-free water/GE Healthcare, #27-4110-01) or in diluted (1:750 in RNAse-free water) tissue lysates from a PBS-treated mouse. The dilution series ranged from 8 ng/μl down to 0.8 zg/μl. A sample containing only 10 ng/μl Poly(A) in RNase-free water was included as NTC and was used to determine the background level of RT-qPCR primers.

### *In vivo* experiments

All experimental animal procedures were approved by the Animal Care and Use Committees of the Kanton Basel, Switzerland.

### Whole tissue lysates preparation

Isolated organs were weighed, transferred into Lysing matrix A tubes (MP Biomedicals, #16910050), frozen on dry ice and stored at −80°C until processing. The tissues were lysed by adding 10 vol/wt Clarity OTX buffer (Phenomenex, #AL0-8498) supplemented with 20 mM Tris(2-carboxyethyl)phosphine (TCEP) (Sigma-Aldrich, #C4706-2G) followed by shaking for 60 s at 4 m/s in a FastPrep-24 instrument (MP Biomedicals). Subsequently, tubes were centrifuged at 4°C for 30 s at 12 000 rpm. Supernatant was transferred to a fresh tube and stored at −80°C. The concentrated lysates were diluted 1:750 in RNase-free water prior to RT-qPCR analysis. Diluted lysates should be stored at −80°C, and repeated freeze-thawing cycles should be avoided.

### Implantation of HCT-116 tumour in mice

The human colorectal carcinoma cell line, HCT-116 (Catalog #CCL-247), was obtained from ATCC (Rockville, Maryland, USA). The cells were maintained and cultured according to established techniques as recommended by the supplier. Briefly, HCT-116 cells were cultivated in McCoy’s 5A medium (Amimed/Bioconcept, #1-18F01-I) supplemented with 10% v/v FCS (Amimed/Bioconcept, #2-01F26-I), 2 mM l-Glutamine (Amimed/Bioconcept, #5-10K00-H) and 2.2 g/L sodium pyruvate (Amimed/Bioconcept, #5-60F00-H). Cells were split 1:8 or 1:10 every third or fourth day depending on confluence. Female Hsd:Athymic nude mice (Harlan Laboratories, Nederland) at age of 6–7 weeks were group housed in individually ventilated cages with access to food and water *ad libitum*. Tumours were established by subcutaneous injection of 5 × 10^6^ cells in 100 µl of HBSS (Invitrogen, #14175-046) per mouse into the left flank of the animals under Isoflurane (Provet, Lyssach, Switzerland) anaesthesia. Tumour volume and body weight were monitored twice weekly. The tumour volume was 429 mm^3^ at the time of sacrifice (38 days after inoculation).

### Mrp4 siRNA QWBA

Male albino CD-1 mouse (Charles River France, 29–32 g) received intravenous administration of unformulated [^3^H]-MRP4 siRNA in 0.9% sodium chloride at a dose of 5 mg/kg, under anaesthesia (by inhalation of an oxygen/isoflurane mixture) as a bolus injection into the *vena saphena*. The dose volume was 2 ml/kg, and the total amount of radioactivity administered was 21 MBq/kg. The mouse was sacrificed by deep isoflurane inhalation at 10 min post *i.v.* dosing. Whole body sections were prepared as described previously and exposed for 2 weeks to Fuji BAS III imaging plates (Fuji Photo Film Co., Ltd., J-Tokyo) in a lead-shielded box at room temperature, and scanned in a Fuji BAS 5000 phosphor imager (Fuji Photo Film) at a 50-µm scanning step.

### AMO-miR-16 injection

Mice were treated intravenously with 80 mg/kg AMO-miR-16 (dissolved in PBS) via tail vein injection. Blood plasma was collected pre- and 5, 10, 30, 60, 240, 360 and 1440 min post dosing. Mice were sacrificed at 1440 min post-dosing and prepared for whole body sectioning or tissue sampling as described previously.

### Whole Body sectioning

Right after sacrifice, animals were submerged in −70°C *n*-hexane (VWR International, #BREN81631-156) / dry ice mixture for about 30 min. The carcasses were stored at −80°C until embedding, and all subsequent procedures were performed at temperatures of about −20°C to minimize diffusion of compound. The frozen carcasses were embedded in a mold on a microtome stage by adding an ice-cold aqueous solution of 2% carboxymethylcellulose (low viscosity, Sigma-Aldrich Chemie GmbH, #C5678-1KG) and freezing it by placement in an *n*-hexane/dry ice bath for about 40 min. The frozen block was removed from the mold and stored at −20°C until sectioning. Whole-body sections were performed in the sagittal plane (from cranial to caudal) using CM3600XP cryomacrotome (Leica Microsystems), and the sections collected on an adhesive tape (Scotch Magic Tape 810, 118 mm × 66 m, Voegtli Bürotechnik AG, #1939899). Several sections of 40-µm thickness were taken at varying depths throughout the body. Sections were mounted on a wooden frame after collection and dehydrated for 72 h at −23°C in the cryomacrotome chamber. The sections were stored at −20°C until further handling.

### WBS-PCR

Firstly, a picture of the section was taken using a ChemiDoc XRS+ System from Bio-Rad. Subsequently, a matrix of 363 wells was outlined on a 1536-well Polypropylene plate (Greiner Bio-One, #789270) with a permanent pen (Staedtler) and each well was filled with 15 μl Clarity OTX buffer (Phenomenex, #AL0-8498) supplemented with 20 mM TCEP (Sigma-Aldrich, #C4706-2G). The whole body section was placed within the matrix on the plate (tissue facing the well) and firmly sealed with a Microseal ‘B’ film (Bio-Rad, #MSB1001) with the help of a rubber brayer (Speedball). Another picture of the section was taken at this point and was used in the data analysis process. The plate was subsequently inversed several times, and centrifuged for 3 min at 1450*g* in a Sigma 4–15C centrifuge (Qiagen) containing a Nr 09100 rotor (Qiagen). Lysates were transferred to a 384-well Hard-Shell PCR Plate (Bio-Rad, #HSP3901) and stored at −80°C. Samples were diluted 1:75 in RNase-free water prior to RT-qPCR analysis. Diluted lysates should be stored at −80°C, and repeated freeze-thawing cycles should be avoided.

### Quantification of rRNA and mRNA

The detection of 18S rRNA and various mRNAs was performed in a one-step reaction. Eight-microlitre RT-qPCR mix containing 5.35 μl DEPC-water, 0.15 μl 100 mM dNTPs (Applied Biosystems, #362271), 1 μl 10× PCR Buffer I (Applied Biosystems, #4379876), 1 μl 25 mM MgCl_2_ (Roche, #12032953001), 0.1 μl Rox Reference Dye (Invitrogen, #12223-012), 0.2 μl Assay On Demand (Applied Biosystems), 0.1 μl Multiscribe (50 U/μl) (Applied Biosystems, #4319983) and 0.1 μl Hot Start Taq Polymerase (5 U/μl) (Roche, #12032953001) was added to a 384-well Hard-Shell PCR Plate (Bio-Rad, #HSP3901) followed by the addition of 2-μl sample. After sealing (Microseal ‘B’ film, Bio-Rad, #MSB1001), the plate was mixed and centrifuged for 3 min at 1450*g*. The PCR reaction was performed in a 7900HT Fast Real-Time PCR System (Applied Biosystems) and consisted of 1 cycle: 30 min/50°C, 1 cycle: 10 min/95°C followed by 40 cycles: 3 s/95°C; 30 s/60°C. The data were acquired and analysed using the software provided by Applied Biosystems. In accordance with Applied Biosystems guidelines, Ct values bigger as 35 were considered as noise.

### Quantification of 18S genomic DNA

The detection of 18S genomic DNA was performed in a one-step reaction. Eight-microlitre qPCR mix containing 5.45 μl DEPC-water, 0.15 μl 100 mM dNTPs (Applied Biosystems, #362271), 1 μl 10× PCR Buffer I (Applied Biosystems, #4379876), 1 μl 25 mM MgCl_2_ (Roche, #12032953001), 0.1 μl Rox Reference Dye (Invitrogen, #12223-012), 0.2 μl Assay On Demand (Applied Biosystems) and 0.1 μl Hot Start Taq Polymerase (5 U/μl) (Roche, #12032953001) was added to each well of a 384-well Hard-Shell PCR Plate (Bio-Rad, #HSP3901) followed by the addition of 2-μl sample. After sealing (Microseal ‘B’ film, Bio-Rad, #MSB1001), the plate was mixed and centrifuged for 3 min at 1450*g*. The PCR reaction was performed in a 7900HT Fast Real-Time PCR System (Applied Biosystems) and consisted of 1 cycle: 10 min/95°C followed by 40 cycles: 3 s/95°C; 30 s/60°C. The data were acquired and analysed using the software provided by Applied Biosystems. In accordance with Applied Biosystems guidelines, Ct values bigger as 35 were considered as noise.

### Quantification of miRNA and siRNA

The detection of miRNAs and siRNAs was performed in a two-step reaction. In the first step, 8 μl RT-mix containing 5.65 μl DEPC-water, 0.15 μl 100 mM dNTPs (Applied Biosystems, #362271), 1 μl 10× PCR Buffer I (Applied Biosystems, #4379876), 1 μl 25 mM MgCl_2_ (Roche, #12032953001), 0.1 μl 1 μM RT-primer and 0.1 μl Multiscribe (50 U/μl) (Applied Biosystems, #4319983) was added to each well of a 384-well Hard-Shell PCR Plate (Bio-Rad, #HSP3901) followed by the addition of 2-μl sample. The plate was sealed (Microseal ‘B’ film, Bio-Rad, #MSB1001), mixed, centrifuged and incubated for 10 min at 25°C followed by 5 min at 95°C in a 7900HT Fast Real-Time PCR System (Applied Biosystems). Before unsealing, the plate was centrifuged for 3 min at 1450*g*, and 5 μl/well PCR mix was added. Subsequently, the plate was sealed (Microseal ‘B’ film, Bio-Rad, #MSB1001), mixed and centrifuged for 3 min at 1450*g*. The PCR mix contained 3.325 μl DEPC-water, 0.15 μl 100 mM dNTPs (Applied Biosystems, #362271), 0.5 μl 10× PCR Buffer I (Applied Biosystems, #4379876), 0.5 μl 25 mM MgCl_2_ (Roche, #12032953001), 0.15 μl 10 μM forward primer, 0.15 μl 10 μM reverse primer, 0.075 μl 50 μM Anti-primer and 0.15 μl Hot Start Taq Polymerase (5 U/μl) (Roche, #12032953001). The PCR reaction was performed in a 7900HT Fast Real-Time PCR System (Applied Biosystems) and consisted of 1 cycle: 10 min/95°C followed by 50 cycles: 3 s/95°C; 30 s/55°C. The ramping speed should not exceed 3°C/s. The data were acquired and analysed using the software provided by Applied Biosystems. Signals that did not fall within the linear range of the standard curve were considered as noise.

### Quantification of AMO-miR-16

The quantification of AMO-miR-16 was performed in a two-step reaction. In the first step, 8 μl Chemical Ligation-mix containing 5.8 μl DEPC-water, 1 μl 10× PCR Buffer (Roche #14882500), 1 μl Poly(A) (1 μg/μl diluted in RNAse-free water/GE Healthcare, #27-4110-01), 0.1 μl 10 μM PS-Ligator (5′-TTAAACCATAGCAGCACG-PS-3′) and 0.1 μl 10 μM BPS-Ligator (5′-BPS-TAAATATTGGCGAACCAGT-3′) was added to each well of a 384-well Hard-Shell PCR Plate (Bio-Rad, #HSP3901) followed by the addition of 2-μl sample. Subsequently, the plate was sealed (Microseal ‘B’ film, Bio-Rad, #MSB1001), mixed, centrifuged for 3 min at 1450*g* and incubated for 30 min at 33°C in a 7900HT Fast Real-Time PCR System (Applied Biosystems). Before unsealing, the plate was centrifuged.

On completion of the chemical ligation reaction, 8 μl PCR-mix containing 6.375 μl DEPC-water, 0.15 μl 100 mM dNTPs (Applied Biosystems, #362271), 1 μl 10× PCR Buffer (Roche #14882500), 0.15 μl 10 μM forward primer, 0.15 μl 10 μM reverse primer, 0.075 μl 50 μM Anti-primer and 0.1 μl Hot Start Taq Polymerase (5 U/μl) (Roche, #12032953001) was added to each well of a new 384-well Hard-Shell PCR Plate (Bio-Rad, #HSP3901) followed by the addition of 2 μl of the Chemical Ligation-mix. Subsequently, the plate was sealed (Microseal ‘B’ film, Bio-Rad, #MSB1001), mixed and centrifuged for 3 min at 1450*g*. The PCR reaction was performed in a 7900HT Fast Real-Time PCR System (Applied Biosystems) and consisted of 1 cycle: 10 min/95°C followed by 50 cycles: 3 s/95°C; 30 s/55°C; 10 s/72°C. The ramping speed should not exceed 3°C/s. The data were acquired and analysed using the software provided by Applied Biosystems. Signals that did not fall within the linear range of the standard curve were considered as noise.

### Statistical analysis

The statistical relevance of the results obtained for the quantification of miR-16 and AMO-miR-16 was tested by Mann–Whitney Rank Sum Test. A *t*-test was performed on the values obtained for miR-191 quantification.

### Imaging

RT-qPCR data of the whole body sections were deconvoluted in Excel and converted into a TissueView compatible image file using an Excel to Analyze Conversion macro (http://maldi.ms). Subsequently, the RT-qPCR image data were loaded into TissueView (AB Sciex, Toronto) and overlaid with an image of the whole body section.

### Building block synthesis for chemical ligators

All reactions were carried out under an atmosphere of argon. Solvents for extraction: technical grade from Brenntag Schweizerhall AG. Solvents for reactions: anhydrous quality from Sigma-Aldrich. Base-protected deoxynucleosides were obtained from ChemGenes (*N*^6^-benzoyl-2′-deoxyadenosine #PM-1101, *N*^4^-benzoyl-2′-deoxycytidine #PM-1102, *N*^2^-isobutyryl-2′-deoxyguanosine #PM-1103, thymidine #DN-1004). All other reagents were purchased from *Sigma-Aldrich*, highest quality available, unless stated otherwise. Flash-chromatography was performed using silica gel 60 with a particle size of 40–63 µm (*Merck*). Thin layer chromatography was performed on *Merck* silica gel 60 *F_254_* plates, which were visualized by UV irradiation (254 nm). NMR: *Bruker DRX-400*, deuterated solvent as indicated, δ in ppm, calibration to residual solvent peaks, ^13^C multiplicities derived from DEPT spectra, ^31^P calibration to external H_3_PO_4_ (δ = 0 ppm).

### Synthesis of base-protected 5′-*o*-biphenylsulphonyl-2′-deoxynucleosides

Five millimoles of the starting base-protected deoxynucleoside 1–4 (Supplementary Figure S13a) is dried by dissolving it twice in 20 ml anhydrous pyridine and evaporating the solvent. The nucleoside is redissolved in 20 ml anhydrous pyridine and cooled to −20°C (4°C for 3) before adding 1.52 g (6.00 mmol) biphenyl-4-sulphonyl chloride. The reaction mixture is stirred for 2 days at −20°C (4°C for 3). After completion, the reaction is quenched by the addition of 5 ml water and stirring is continued for 15 min at room temperature. The reaction mixture is then taken up in 100 ml dichloromethane and washed with saturated aqueous sodium bicarbonate solution (2 × 50 ml) and brine (50 ml). The aqueous layers are washed twice with 50 ml dichloromethane each. The combined organic phases are dried over anhydrous sodium sulphate, filtered and evaporated. Residual pyridine is removed by repeated co-evaporation with toluene (3 × 10 ml). The crude product is purified by flash chromatography yielding the 5′-*o*-biphenylsulphonyl-2′-deoxynucleoside 5–8 as off-white foam. Analytical data see Supplementary Table S7.

### Synthesis of N-protected 5′-*o*-biphenylsulphonyl-2′-deoxynucleoside phosphoramidites

To a solution of 5.00 mmol of the N-protected 5′-*o*-biphenylsulphonyl-2′-deoxynucleoside 5–8 in 25 ml anhydrous dichloromethane under argon is added 1.28 g (7.50 mmol) diisopropylammonium-tetrazolide (prepared from tetrazole and diisopropylamine or from Chem-Impex, #00951). Then 2.22 ml (2.11 g, 7.00 mmol) 2-cyanoethyl N,N,N',N'-tetraisopropylphosphordiamidite (Aldrich 97%, #305995) is added, and the solution stirred at room temperature overnight. The reaction mixture is taken up in 100 ml dichloromethane and extracted with aqueous bicarbonate solution (2 × 50 ml) and brine (50 ml). The aqueous phases are reextracted with 50 ml dichloromethane. The combined organic phases are dried over sodium sulphate, filtered and evaporated. The remaining oil is chromatographed on silica to give a mixture of diastereomeric 5′-*o*-biphenylsulphonyl-2′-deoxynucleoside phosphoramidites 9–12 (Supplementary Figure S13b) as colourless brittle foam. Analytical data see Supplementary Table S7.

### Synthesis of oligodeoxynucleotides with 3′-terminal phosphorothioate

DNA oligonucleotides were prepared on a MerMade 192 well DNA/RNA Synthesizer (BioAutomation) on a scale of 200 nmol. TheraPure™ deoxyribonucleoside phosphoramidites were dissolved in anhydrous acetonitrile at a concentration of 0.1 M. The activator, 5-ethylthiotetrazole (ChemGenes, #RN-6397), was dissolved in acetonitrile as a 0.5 M solution. Capping solution A consisted of acetic anhydride (Fluka, #45830) and 2,6-lutidine (Aldrich, #336106) in anhydrous THF (Aldrich, #401757) at a ratio of 1:1:8 (v/v/v) and capping solution B of 16% (v/v) 1-methylimidazole (Aldrich, #336092) in anhydrous THF. The oxidation solution was prepared by dissolving iodine (Aldrich, #207772) in a 1:1:8 (v/v/v) mixture of 2,6-lutidine, water and THF for a final concentration of 0.1 M. Deblocking solution was 4% (v/v) dichloroacetic acid (Aldrich, #D54702) in dichloromethane (Aldrich, #270997). Sulphurizing reagent consisted of a 0.05 M solution of 3 -((dimethylaminomethylidene)amino)-3H-1,2,4-dithiazole-3-thione (Sulphurizing Reagent II, Glen Research, #40-4037-20) in pyridine/acetonitrile 3:2 (v/v). The synthesis cycle consisted of the following steps: (i) detritylation with deblocking solution (2 × 90 μl, 10 s each), wash with acetonitrile (2 × 100 μl); (ii) coupling reaction with a 6:1 (v/v) mixture of activator and phosphoramidite solution (2 × 35 μl, 3 min each), wash with acetonitrile (2 × 100 μl); (iii) capping with a 1:1 (v/v) mixture of capping solutions A and B (100 μl, 20 s), wash with acetonitrile (2 × 100 μl); (iv) oxidation with iodine solution (100 μl, 60 s), wash with acetonitrile (2 × 100 μl). Reagents are applied to the top of the columns and allowed to react for the time indicated. For their removal, a gentle vacuum is applied as per manufacturer’s instructions. Oligodeoxynucleotides are synthesized without removing the 5′-terminal dimethoxytrityl group. For the preparation of DNA oligonucleotides with a 3′-terminal thiophosphate, a prepacked 200 nmol synthesis column (BioAutomation) was emptied and charged with 5 mg of 3′-phosphate CPG (Glen Research, #20-2900-01) (12). The following cycle is applied in the first coupling: (i) detritylation with deblocking solution (2 × 90 μl, 10 s each), (ii) coupling reaction with a 6:1 (v/v) mixture of activator and phosphoramidite solution (2 × 35 μl, 3 min each), wash with acetonitrile (2 × 100 μl); (iii) sulphurization with sulphurizing reagent II (100 μl, 60 s) wash with acetonitrile (2 × 100 μl); (iv) capping with a 1:1 (v/v) mixture of capping solutions A and B (100 μl, 20 s), wash with acetonitrile (2 × 100 μl). The oligodeoxynucleotides were detached from solid support and deprotected by treating with concentrated ammonia (Merck, #1.05426.1000) at 55°C overnight. The ammonia solution was filtered off, and the resin washed with 500 μl water. The filtrate was lyophilized and the residue redissolved in 1 ml aqueous 0.1 M triethylammonium acetate buffer pH 7.0. The crude oligodeoxynucleotide was purified on a Oasis HLB 96-well plate (30-μm particle size, Waters, #WAT058951) applying the following protocol: (i) each well used is equilibrated by washing with 500 μl acetonitrile and 1 ml aqueous 0.1 M triethylammonium acetate buffer pH 7.0; (ii) the oligodeoxynucleotide solution above is applied to a single well; (iii) the resin is washed with 1 ml 15% acetonitrile/water (v/v); (iv) the DMT group is removed by applying 1 ml 3% aqueous dichloroacetic acid; (v) wash with 2 × 1 ml aqueous 0.1 M triethylammonium acetate buffer pH 7.0; (vi) column is sucked dry by applying a gentle vacuum from the bottom; (vii) apply 530 μl acetonitrile/water 1:1 (v/v) and collect oligonucleotide. The oligodeoxynucleotide solutions were evaporated on a plate lyophilizer (GeneVac, HT-4×), and the residue redissolved in water for a 80 μM stock solution. Purity and identity of all oligonucleotides were verified by LC-MS analysis (Thermo Scientific Accela 600 pumps, Accela PDA detector, and LCQ Fleet MS, equipped with a PAL System HTS PAL autosampler).

### Synthesis of 5′-*o*-biphenylsulphonyl-derivatized oligodeoxynucleotides

Oligodeoxynucleotides containing a 5′-*o*-biphenylsulphonyl group were synthesized at a scale of 200 nmol as described above, except that Ultramild CE phosphoramidites (http://www.glenresearch.com/Technical/TB_UltraMild_Deprotection.pdf) for regular deoxynucleosides were used and for the final coupling step phosphoramidites 9–12. Capping was performed with phenoxyacetic anhydride (Aldrich, order no. 77750) substituting acetic anhydride. For deprotection, the CPG was treated with concentrated ammonia for 4 h at room temperature. Purification was achieved as above on Oasis HLB reverse phase cartridges, except that steps (iv) and (v) were omitted.

### Oligonucleotide synthesis

Phosphoramidites for 200–800 μmol syntheses of fully 2′-*o*-methyoxyethyl-modified oligoribonucleotides were custom synthesized (13). Control pore glass (CPG) solid supports with preloaded 2′-*o*-methyoxyethyl ribonucleoside monomers were custom synthesized starting from commercially available amine-modified CPG (Prime Synthesis, LCAA/CNA CPG, #CPG601N12). Ultramild CE phosphoramidites for 200 nmol syntheses of 5′-*o*-biphenylsulphonyl-modified oligodeoxynucleotides were purchased from Glen Research (Pac-dA-CE Phosphoramidite #10-1601-10, Ac-dC-CE Phosphoramidite #10-1015-10, iPr-Pac-dG-CE Phosphoramidite #10-1621-10). TheraPure™ DNA phosphoramidites were purchased from Thermo Scientific (Bz-dA-CE Phosphoramidite #27-2030-00, Bz-dC-CE Phosphoramidite #27-2032-00, iBu-dG-CE Phosphoramidite #27-2034-00, dT-CE Phosphoramidite #27-2036-00). Support for DNA oligonucleotides with a 3′-thiophosphate was obtained from Glen Research (3′-Phosphate CPG #20-2900-10). Anhydrous acetonitrile for oligonucleotide synthesis was purchased either from Biosolve (#012058, <10 ppm water) or from Sigma Aldrich (#L0114000-01, <30 ppm water). Sterile water for buffers and oligonucleotide solutions was obtained from a Milli-Q Advantage A10 station (Millipore).

### AMO-miR-16 synthesis

The base-protected 2′-*o*-methoxyethyl ribonucleoside phosphoramidites were dissolved in anhydrous acetonitrile at a concentration of 0.15 M. AMO-miR-16 synthesis was performed on an Äkta Oligopilot plus OP100 (GE Healthcare) with the help of a 12-ml column reactor (GE Healthcare, #18-1101-16) using solid-phase synthesis cyclic procedure consisting of five steps, based on the manufacturer’s template method. First, the 4,4′-dimethoxytrityl (DMT) protecting group was removed by adding deblocking reagent (3% DCA in toluene, Merck, #BI0832). Coupling was then achieved by adding the n+1 phophoramidite solution to the column along with an equal volume of 0.5 M 5-ethylthiotetrazole (ChemGenes, #RN-6397) in acetonitrile. Coupling was followed by capping by adding Cap A (Biosolve, #036124) and Cap B, obtained by mixing equivalent amounts of Cap B1 (Biosolve, #037424) and Cap B2 (Biosolve, #037425). The oligonucleotide was subsequently oxidized by adding iodine (Biosolve, #150724). The column was washed between each step with anhydrous acetonitrile. The synthesis procedure ended with a final detritylation step in order to remove the last DMT group and allow easier purification. After synthesis the column was placed under vacuum to remove all remaining acetonitrile, and the support was transferred into a glass vial. The oligonucleotide was cleaved from the support and deprotected by incubating the support with 60-ml conc. ammonia solution 32% (Merck, #105426) overnight at 50°C under agitation. The solution was subsequently filtered on a Steritop filter unit (Millipore, #SCGPT05RE) and the filter was rinsed with water. The oligonucleotide was purified on a Fineline pilot 35 column (GE Healthcare, #18-1102-02) filled with TSK Gel SuperQ-5PW ion exchange resin (Tosoh, #18546) using an ÄKTAexplorer (GE Healthcare). Fractions of appropriate purity were pooled and then desalted by tangential flow filtration (TFF) on a Minim II TFF system (Pall Corporation) using a 1 K Omega Centramate T-Series filtration unit (Pall Corporation, #OS001T12). The oligonucleotide was filtered again on a Steriflip-GP filtration unit (Millipore, #SCGP00525) and the quality of the oligonucleotide verified by HPLC and UPLC-MS. Prior to injection into animals, endotoxin levels were assessed using the Endosafe-PTS system (Charles River) and PTS cartridges (Charles River, #PTS2005).

## RESULTS

### Validation of the WBS-PCR using endogenously expressed mRNAs

The WBS-PCR procedure (Supplementary Figure S1) makes use of 40-μm sagittal cross-sections of a mouse, typically generated for biodistribution studies of novel radiolabelled therapeutic compounds involving Quantitative Whole-Body Autoradiography (QWBA) (14), the spatial resolution of a 1536 round-well plate and a potent tissue lysis buffer. By placing the mouse section on a pre-filled 1536-well plate and extracting the target from the exposed tissues by simply inverting the plate, the entire section is deconvoluted into separate tissue lysates, which can be subjected to down-stream analytical assays such as RT-qPCR. Typically, a mouse section covers ∼363 wells of the 1536-well plate which, when analyzed on a 384-well qPCR plate, reserves 20 wells for the inclusion of reference samples or standards. Subsequently, with the use of spreadsheets (i.e. Excel) and imaging software (i.e. Tissue View), the localization of the analyte is visualized by converting the qPCR signals into an image and overlaying them with a picture taken of the whole-body cross-section prior to the extraction procedure.

**Figure 1.**
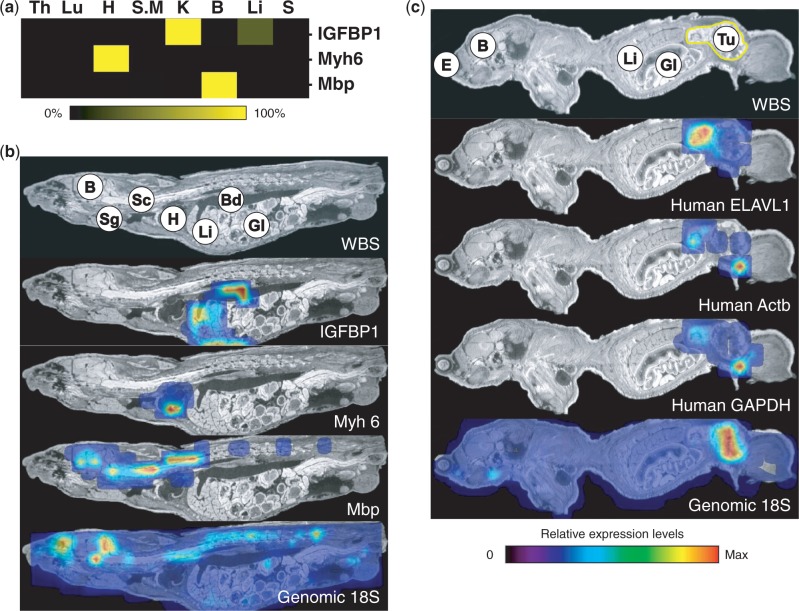
Biodistribution of endogenous and exogenous tissue-specific mRNAs. (**a**) The compatibility of the tissue lysis buffer was validated by characterizing the expression profiles of tissue-specific mRNAs: Insulin-like Growth Factor-Binding Protein 1 (IGFBP1), Myosin heavy chain 6 (Myh6) and Myelin Basic Protein (Mbp) in lysates prepared from Thymus (Th), Lung (Lu), Heart (H), Skeletal muscle (S.M), Kidney (K), Brain (B), Liver (Li) and Spleen (S). Signals were normalized against genomic 18S and the maximum averaged signal for each mRNA was set to 100%. (**b**) WBS-PCR biodistribution of the mRNAs characterized in (a). WBS (upper panel) represents the whole body section before lysis. Tissue annotation: Brain (B), Spinal Cord (Sc), Salivary gland (Sg), Heart (H), Liver (Li), Blood (Bd) and Gastro-Intestinal tract (GI). All signals were normalized against genomic 18S (Lower panel). (**c**) WBS-PCR biodistribution of human embryonic lethal, abnormal vision, Drosophila-like 1 (ELAVL1), human Actin Beat (Actb) and glyceraldehyde 3-phosphate dehydrogenase (GAPDH) mRNAs in a HCT-116 human tumour-bearing mouse. Tissue annotation in the WBS: Eye (E), Brain (B), Liver (Li), Gastro-Intestinal tract (GI) and HCT-116 tumour (T). All signals were normalized against genomic 18S (Lower panel).

Because different endogenous RNAs and oligonucleotides (i.e. mRNA, miRNA, siRNA and antagomir) require different purification methods for their optimal isolation from tissues, we investigated the possibility to measure their levels directly in tissue lysates without the need for extensive purification steps. This approach minimizes the loss of signal intensity owing to biased purification procedures and allows for high through-put sample analysis using liquid handling robotics. Another advantage of this approach is that the lysates contain endogenous genomic DNA, which can be used to normalize the mRNA signals obtained in the RT-qPCR (15,16) without the need to analyse large panels of housekeeping genes (17). A potential disadvantage of this approach, on the other hand, is that most reagents used for preparing tissue lysates contain potent protein denaturants, high salt concentrations and chelating agents, which could inhibit or reduce the efficiency of the enzymatic reactions required for quantification (18). However, by taking advantage of the sensitivity of typical RT-qPCR based assays, the use of these reagents could be permitted by incorporating a dilution step to reduce or eliminate the inhibitory elements introduced during the extraction procedure. We have tested several lysis buffers and found that the Clarity OTX lysis buffer was the most compatible with RT-qPCR. To demonstrate this compatibility, we established the expression profiles of a small panel of tissue-specific mRNAs obtained by subjecting the diluted tissue lysates (Figure 1a, Supplementary Table S1) directly into the RT-qPCR reaction. We also measured the levels of genomic 18S in the lysates by qPCR and used these internal reference signals to normalize the obtained mRNA signals. The compatibility of the tissue lysis buffer with RT-qPCR was confirmed with several specific expression profiles: Insulin-like growth factor-binding protein 1 (IGFBP-1) mRNA was limited to liver and kidney (19), Myosin heavy chain 6 (Myh6) mRNA could only be detected in lysates prepared from heart and lung (20) and the mRNA encoding Myelin basic protein (Mbp) (21) could exclusively be detected in the brain. As expected, similar expression patterns were observed when RT-qPCR reactions were performed using purified total RNA (Supplementary Figure S2, Supplementary Table S2).

As a next step, the biodistribution of the same panel of mRNA targets was investigated using the WBS-PCR method (Figure 1b). The efficiency of the extraction procedure was monitored by measuring genomic 18S signals across the whole body section (Figure 1b, lower panel). As expected, genomic 18S displayed an overall homogeneous biodistribution pattern with some elevated levels observed in the brain. These elevated levels could be due to variations in tissue cell-densities and extraction efficiency. To compensate for these potential biases, all WBS-PCR signals are corrected against genomic 18S. Unlike genomic 18S, the mRNA targets displayed more distinct biodistribution patterns that corroborated the expression profiles obtained for the tissue homogenates in all cases. The biodistribution pattern obtained for IGFBP-1 mRNA co-localized with the liver, whereas Myh6 mRNA signals co-localized only with the heart region. Interestingly, the Mbp biodistribution pattern confirmed the expression of mRNA in brain but interestingly also in regions along the spinal cord. Intrigued with this observation, the RT-qPCR reaction was repeated on purified total RNA (Supplementary Figure S2 and Supplementary Table S2), and the presence of Mbp mRNA in both brain and spinal cord could be confirmed. This observation illustrates the power of the WBS-PCR in visualizing the biodistribution of uncharacterized mRNAs.

To further validate the optical resolution that could be achieved with the WBS-PCR, we investigated the biodistribution pattern of human mRNAs encoding ELAVL1 (embryonic lethal, abnormal vision, Drosophila-like 1), Actb (Beta actin) and GAPDH (glyceraldehyde-3-phosphate dehydrogenase) in a section obtained from a human tumour (HCT-116)-bearing mouse (Figure 1c) using human specific RT-qPCR primers. As expected, the biodistribution pattern for the human mRNAs co-localized exactly with the region of tumour growth. Because the 18S primers recognize both human and mouse sequences, genomic 18S could be detected across the section with elevated levels in the tumour region (Figure 1c, lower panel). Taken together, these data indicate that the deconvolution of a whole-body mouse section into 363 separate samples followed by the re-assembly of the RT-qPCR signals into an image can generate accurate interpretable biodistribution data and may provide more detailed expression information than can be derived from individually isolated tissues.

### Whole body scanning PCR for the localization of miRNAs and siRNAs

**Figure 2.**
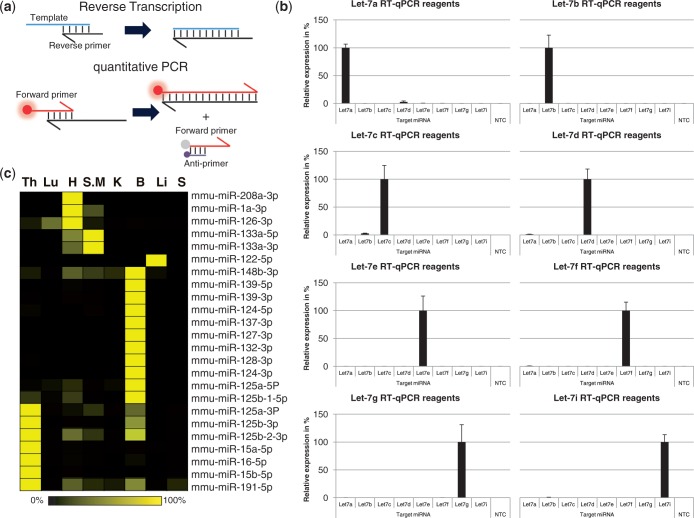
Characterization and validation of the miRNA RT-qPCR method. (**a**) RT-qPCR assay for miRNAs and siRNAs. The RT/Reverse primer is used to generate a cDNA copy of the RNA template during the Reverse Transcription step. Real-time fluorescent quantification occurs during the qPCR step with the help of a fluorescently labelled Forward primer hybridizing to the elongated RT/Reverse primer. Signals generated by non-incorporated forward primers are quenched by a quencher-labelled anti-primer. (**b**) The specificity of the RT-qPCR method was verified by analysing the back-ground signal intensity generated by let-7 miRNA-specific primers using either Let-7a, -7b, -7c, -7d, -7e, -7f, -7g, -7i as template in the reaction. Average values for the perfectly matched targets were set at 100%. Error bars, STDEV (*n* = 4). (**c**) miRNA expression profile in diluted tissue homogenates: Thymus (Th), Lung (Lu), Heart (H), Skeletal Muscle (S.M), Kidney (K), Brain (B), Liver (Li) and Spleen (S). Signals were normalized against genomic 18S, and the maximum averaged signal for each mRNA was set to 100%.

The RNA biodistribution data reported above were obtained using the highly specific TaqMan assay, which relies on the specific amplification of a target gene using two primers and the hydrolysis of a TaqMan probe. Although similar probe-based assays are described for the detection of siRNAs (22,23), we set out to develop an RT-qPCR assay with the aim of reducing the amount of sample handling steps without compromising assay specificity or sensitivity (Figure 2a). Like most assays, the first step relies on the classical conversion of RNA into cDNA by reverse transcriptase-mediated primer extension (24). In the second step, a qPCR-mix is directly added to the heat-inactivated reverse-transcription reaction followed by an anti-primer quenching-based real-time PCR reaction (25). Real-time fluorescent quantification takes place during the extension cycle of the PCR reaction. During this cycle, the un-incorporated forward primers will be quenched by the quencher-labelled anti-primer whereas forward primers that constitute the double-stranded PCR products will not. An exponentially increasing fluorescent signal will be the result of successful target amplification. Because miRNAs and siRNAs are similar in size and hence pose similar challenges with respect to designing reliable primers, the specificity of the assay was evaluated in a typical matrix study whereby the level of cross-reactivity was measured against a panel of closely related Let-7 miRNA family members (Figure 2b). Taken into account that the perfectly matched target was arbitrarily set at 100%, the highest cross-reactivity (∼3%) was observed only when Let-7d and Let-7b levels were determined using primers designed against Let-7a or Let-7c, respectively. All other template/primer combinations resulted in minimal cross-reactivity ranging between 0 and 1%. Similar levels of specificity were reported using commercially available TaqMan miRNA assays (26). In addition to this, all the Let-7 assays displayed a large dynamic range (8–10 logs) with a lower limit of detection ranging from 0.02 to 0.002 femtogram, highlighting the level of sensitivity that can be achieved using this RT-qPCR assay (Supplementary Figure S3). The performance of the RT-qPCR assay was further characterized by measuring the relative expression levels of a panel of miRNAs in the same mouse organ homogenates described previously (Figure 2c and Supplementary Table S3). As expected, detection of the well characterized heart- and muscle-specific miRNAs (e.g. miR-208a, miR-1a, miR-133a), liver-specific (e.g. miR-122) and brain-enriched miRNAs (e.g. miR-124, miR-127, miR-128, miR-132, miR-137, miR-139) in the appropriate organs (27–29) confirms the compatibility of our method with measuring miRNAs directly in tissue lysates. In addition to this, RT-qPCR analysis of miRNA 122-5p, 208a-3p, 124-3p, 124-5p, 191-5p and 16-5p using total RNA as input in the reaction resulted in similar expression profiles (Supplementary Figure S4 and Supplementary Table S4). Taken together, the data strongly suggest that the lysis procedure is indeed compatible with the specific detection of tissue-derived miRNAs.

**Figure 3.**
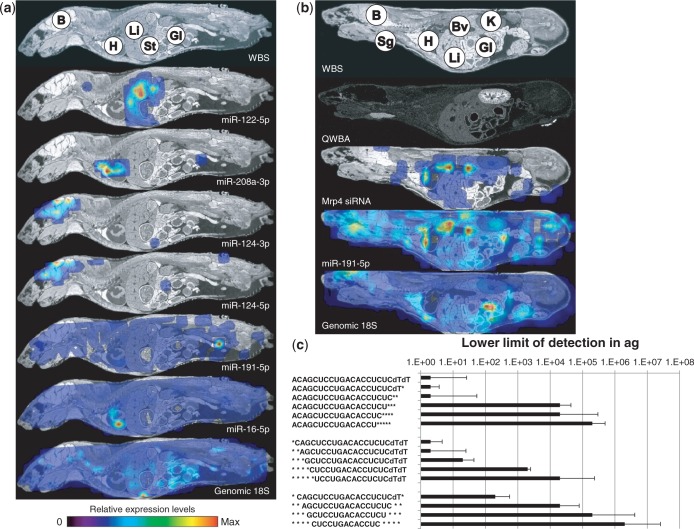
Biodistribution of endogenous tissue-specific miRNAs and an exogenous tritium-labelled siRNA. (**a**) Biodistribution of tissue-specific miRNAs using WBS-PCR. Tissue annotation: Brain (B), Heart (H), Liver (Li), Stomach (St) and Gastro-Intestinal tract (GI). All signals were normalized against genomic 18S (Lower panel). (**b**) Biodistribution of tritium-labelled unformulated siRNA targeting Mrp4 by phosphor imaging (QWBA) and WBS-PCR (Mrp4 siRNA) at 10 min post dosing (the whiter the area in the autoradiograms using QWBA, the higher the concentration of radioactivity) as well as the WBS-PCR visualization of the biodistribution obtained for miR-191. All miRNA and siRNA signals were normalized against genomic 18S (Lower panel). (**c**) Detection limit of the RT-qPCR assay using 3′-truncated (upper panel), 5′-truncated (middle panel) and 3′/5′-truncated (lower panel) Mrp4 anti-sense strands as template in the reaction.

The specificity of the RT-qPCR assay was further supported by the biodistribution patterns obtained for the tissue-specific miRNAs miR-122 (liver), miR-208a (heart) and miR-124-3p/-5p (brain), and the ubiquitously expressed miRNAs miR-191 and miR-16, using the WBS-PCR method (Figure 3a). Again, genomic 18S was used to confirm extraction efficiency and to normalize all miRNA signals (Figure 3, lower panel). Interestingly, the expression of miR-124-3p and miR-124-5p, both processed from the same pre-miRNA, displayed very similar brain-specific biodistribution patterns.

As a more stringent test, we compared the biodistribution pattern of the WBS-PCR method with the traditional quantitative whole-body autoradiograph (QWBA) obtained from a mouse intravenously dosed with an unformulated, tritium labelled siRNA targeting the rat multidrug resistance protein 4 (Mrp4 siRNA) (10) (Figure 3b and Supplementary Figure S5). Analysis of a mouse section, sacrificed 10 min post intravenous dosing, revealed that the high levels of total radiolabelled components observed in kidney and liver, represented by the white regions in the autoradiograms using QWBA, were also present in the biodistribution pattern obtained using the WBS-PCR method. Despite the presence of radioactivity in the salivary gland, the WBS-PCR failed to confirm the presence of siRNA in this organ. Lack of siRNA RT-qPCR signal could not be ascribed to poor extraction efficiency due to the clear presence of both genomic 18S and miR-191 in this region. Indeed, LC-MS analysis confirmed that the radioactive signal observed in the salivary gland did not represent that of intact siRNA but rather that of the tritiated metabolite mono-Uridine (data not shown). To investigate the limitation of the RT-qPCR with respect to detecting metabolites, we generated various truncations of the anti-sense strand of the siRNA duplex, spiked them into rat total RNA and determined for each template the lowest amount of target that could still be detectable by RT-qPCR (Figure 3c). As expected, deletion of more than two nucleotides at either the 5′ or 3′ end of the target sequence results in a rapid decrease in sensitivity of the RT-qPCR assay. Hence, metabolites such as mono-Uridine would also not be detected by RT-qPCR. Taken together; these data confirm that the signals obtained in the WBS-PCR originate from either full length RNA or minimally truncated metabolites. Additionally, lack of detection of metabolites, such as mono-Uridine, provides a signal specificity, whereas the classical QWBA approach provides the detection of total radioactivity from parent compounds and metabolites.

### Whole body scanning PCR for the localization of chemically modified oligonucleotides

**Figure 4.**
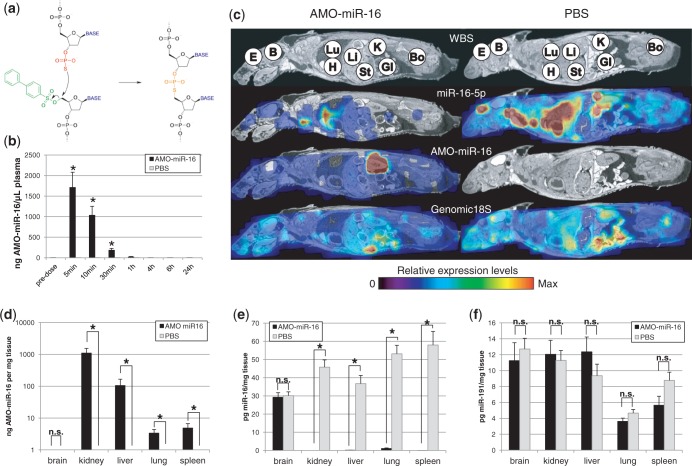
Validation of the biodistribution obtained for AMO-miR-16 using CL-qPCR. (**a**) Chemical reaction that occurs during the side-by-side target hybridization of a DNA-oligo containing a 3′-phosphorothioate group and a DNA-oligo containing a 5′-biphenylsulphonlyl group. The template-mediated, proximity-dependent reaction results in displacement of the biphenylsulphonyl leaving group and the formation of a carbon–sulphur bond. (BASE represents Adenosine, Guanosine, Thymidine or Cytidine). (**b**) Quantification of AMO-miR-16 in plasma isolated from AMO-miR-16- (black) and PBS-treated (grey) mice. Values are the average of triplicate measurements from five animals. Error bars, s.e.m (*n* = 15). *P < 0.05 (Mann–Whitney Rank Sum test). (**c**) The biodistribution of miR-16 and AMO-miR-16 were determined in mouse whole body sections, treated either with AMO-miR-16 (left panel) or with PBS (right panel) and normalized against genomic 18S. Tissue annotation: Eye (E), Brain (B), Lung (Lu), Heart (H), Liver (Li), Stomach (St), Kidney (K), Bone (Bo) and Gastro-Intestinal tract (GI). (**d–f**) Quantification of AMO-miR-16 (d), miR-16 (e) and miR-191 (f) in tissues isolated from mice treated either with AMO-miR-16 (black) or PBS (grey). Values are the average of triplicate measurements from four animals. Error bars, s.e.m. (*n* = 12). *P < 0.05 [Mann–Whitney Rank Sum test (d and e), *t*-test (f), n.s.: not significant].

Most therapeutic oligonucleotides contain various degrees of chemical modifications to confer appropriate characteristics such as nuclease resistance, affinity, specificity, safety, distribution and cellular uptake. Although chemical modifications can improve the pharmacokinetic and dynamic properties of these oligonucleotides, they also pose analytical challenges. For example, a 2′-*o*-Methyl-modified anti-miRNA-16 oligonucleotide (AMO-miR-16) is readily detectable by RT-qPCR (30) whereas a 2′-*o*-(2-methoxyethyl)-modified sequence is not (Supplementary Figure S6). For this reason, we developed the CL-qPCR assay; a two-step assay that uses qPCR to quantify the amount of product formed in a self-directed chemical-ligation of two oligodeoxynucleotides templated by the fully complementary analyte (11,31) (Supplementary Figure S7a), independently of the sequence (Supplementary Figure S7b) or the chemical modifications present in the template (Supplementary Figure S8). During the first step, binding of the two DNA oligonucleotides, side-by-side, on the target initiates the reaction of the nucleophilic phosphorothioate group, located on the 3′-end of one oligonucleotide, with the electrophilic carbon at the 5′-end of the adjacent oligonucleotide. This proximity-dependent reaction results in displacement of the sulphonate leaving group and the formation of a carbon–sulphur bond, covalently linking the two oligonucleotides into a single unique DNA-oligonucleotide (Figure 4a), which can be quantified by PCR, despite the carbon–sulphur bond being somewhat longer than the carbon–oxygen bond of regular DNA.

Next, we investigated the biodistribution of the MOE-modified AMO-miR-16 using CL-qPCR. For this, two groups of mice, each consisting of five animals, were dosed intravenously with either AMO-miR-16 (80 mg/kg) or PBS. Plasma samples were collected at various time points over a period of 24 h, and AMO-miR-16 levels were quantified using the CL-qPCR method. As expected, AMO-miR-16 was rapidly cleared from the plasma reaching undetectable levels within 1 h post dosing (Figure 4b and Supplementary Figure S9). Despite the rapid plasma clearance, WBS-PCR on sections obtained 24 h post dosing revealed a broad biodistribution pattern of AMO-miR-16, suggesting extensive uptake of AMO-miR-16 in a wide variety of tissues (Figure 4c). The highest levels of AMO-miR-16 could be observed in the kidney whereas no signal could be detected in samples co-localizing with the brain. Interestingly, a 20-mer MOE-modified PO oligonucleotide targeting human intercellular adhesion molecule-1 mRNA dosed in rats displays a similar plasma pharmacokinetic profile and biodistribution pattern, confirming the performance of the CL-qPCR (32). In addition to this, dosing of AMO-miR-16 lowered the global biodistribution levels of miR-16 in the AMO-miR-16-treated animal compared with those in the PBS-treated animal. The biodistribution of genomic 18S, on the other hand, was very similar between the two animals and did not suggest that lack of miR-16 could be due to poor extraction efficiencies. To confirm the WBS-PCR observation, AMO-miR-16 (Figure 4d and Supplementary Figure S10) and miR16 levels (Figure 4e and Supplementary Figure S11) were measured in the organs isolated from the remaining four animals. Indeed, quantification of AMO-miR-16 by CL-qPCR confirmed the presence of compound in the kidney, liver, lung and spleen but not in the brain of the treated animals. The presence of AMO-miR-16 also significantly reduced miR-16 levels in these tissues whereas miR-191 levels (Figure 4f and Supplementary Figure S12) remained unaffected, suggesting that the loss of miR-16 could indeed be the result of AMO binding. Taken together, these data confirm both the robustness of the CL-qPCR method in the quantification of heavily chemically modified oligonucleotides in biological samples and its application in biodistribution studies using WBS-PCR.

## DISCUSSION

The therapeutic potential of oligonucleotides has been convincingly realized in many different *in vitro* and preclinical disease models. However, the transition from bench to bed-side is hampered by the same recurring issue, namely safe and efficient delivery to specific tissues. One of the many strategies to improve the therapeutic window is to enhance the homing or tissue-specific uptake by incorporation of ligands such as peptides, antibodies, natural products and aptamers into the delivery vehicle or by direct conjugation to the oligonucleotides (2,33). With the use of *in vitro* reporter-based cell assays, it is fairly straightforward to establish screening platforms that can be used in the search for ligands with particular characteristics such as cell penetration or receptor-mediated uptake. However, there is a risk that candidates isolated via an *in vitro* selection may loose efficiency when applied *in vivo*. WBS-PCR offers a unique *in vivo* screening platform to search for those moieties that are able to improve the delivery efficiency of therapeutic oligonucleotides. Because the imaging procedure is based on the detection of the therapeutic oligonucleotide by either RT-qPCR or CL-qPCR, there is no limitation with respect to the chemical formats of the oligonucleotides nor is there a need to include radioactivity or tracer units such as fluorescent dyes or any other agents that might influence the delivery characteristics. The advantages of the assay go further as it visualizes predominantly the localization of intact therapeutic oligonucleotides. This level of specificity could prove valuable in reducing the false-positive hits caused by the accumulation of metabolites in secondary tissues. Clearly, WBS-PCR would not provide the inter- and intra-cellular resolution that can be obtained with more traditional assays such as *in situ* hybridization. However, the obtained resolution is enough to visualize distinct biodistribution patterns that can be assigned to various tissues within the whole body section. In addition to this, the simplicity of the assay allows for a rapid and cost-effective screening method, which can be totally automated. This is an indispensible characteristic when considering the pressures associated with drug development.

The method described here reports the use of WBS-PCR in visualizing the biodistribution of a single oligonucleotide-based target. One of the future challenges will be to multiplex this assay by dosing and analysing several compounds simultaneously, thereby increasing the screening throughput. In addition, the combination of WBS-PCR imaging with aptamer technology could lead to novel exciting applications such as the whole body biodistribution of peptides and proteins (34–37).

## SUPPLEMENTARY DATA

Supplementary Data are available at NAR Online: Supplementary Tables 1–7 and Supplementary Figures 1–13.

## FUNDING

All authors are employees of Novartis, Inc. Funding for open access charge: Novartis Pharma AG.

*Conflict of interest statement*. None declared.

## Supplementary Material

Supplementary Data

## References

[1] Xu L, Anchordoquy T (2011). Drug delivery trends in clinical trials and translational medicine: challenges and opportunities in the delivery of nucleic acid-based therapeutics. J. Pharma. Sci..

[2] Yuan X, Naguib S, Wu Z (2011). Recent advances of siRNA delivery by nanoparticles. Expert Opin. Drug Deliv..

[3] Lennox KA, Behlke MA (2011). Chemical modification and design of anti-miRNA oligonucleotides. Gene Ther..

[4] Behlke MA (2008). Chemical modification of siRNAs for *in vivo* use. Oligonucleotides.

[5] Tavitian B (2003). *In vivo* imaging with oligonucleotides for diagnosis and drug development. Gut.

[6] Lionnet T, Czaplinski K, Darzacq X, Shav-Tal Y, Wells AL, Chao JA, Park HY, de Turris V, Lopez-Jones M, Singer RH (2011). A transgenic mouse for *in vivo* detection of endogenous labeled mRNA. Nat. Methods.

[7] Medarova Z, Pham W, Farrar C, Petkova V, Moore A (2007). *In vivo* imaging of siRNA delivery and silencing in tumors. Nat. Med..

[8] Rhee WJ, Bao G (2010). Slow non-specific accumulation of 2'-deoxy and 2'-O-methyl oligonucleotide probes at mitochondria in live cells. Nucleic Acids Res..

[9] Yoo H, Juliano RL (2000). Enhanced delivery of antisense oligonucleotides with fluorophore-conjugated PAMAM dendrimers. Nucleic Acids Res..

[10] Christensen J, Natt F, Hunziker J, Krauser J, Andres H, Swart P (2012). Tritium labeling of full-length small interfering RNAs. J. Labelled Comp. Radiopharm..

[11] Silverman AP, Kool ET (2006). Detecting RNA and DNA with templated chemical reactions. Chem. Rev..

[12] Horn T, Urdea MS (1986). A chemical 5'-phosphorylation of oligodeoxyribonucleotides. DNA.

[13] Martin P (1995). A new access to 2′-O-Alkylated Ribonucleosides and properties of 2'-O-Alkylated Oligoribonucleotides. Helv. Chim. Acta.

[14] Coe RA (2000). Quantitative whole-body autoradiography. Regul. Toxicol. Pharmacol..

[15] Kanno J, Aisaki K, Igarashi K, Nakatsu N, Ono A, Kodama Y, Nagao T (2006). “Per cell” normalization method for mRNA measurement by quantitative PCR and microarrays. BMC Genomics.

[16] Talaat AM, Howard ST, Hale WT, Lyons R, Garner H, Johnston SA (2002). Genomic DNA standards for gene expression profiling in Mycobacterium tuberculosis. Nucleic Acids Res..

[17] Vandesompele J, De Preter K, Pattyn F, Poppe B, Van Roy N, De Paepe A, Speleman F (2002). Accurate normalization of real-time quantitative RT-PCR data by geometric averaging of multiple internal control genes. Genome Biol..

[18] Chomczynski P, Sacchi N (2006). The single-step method of RNA isolation by acid guanidinium thiocyanate-phenol-chloroform extraction: twenty-something years on. Nat. Protoc..

[19] Crissey MA, Leu JI, DeAngelis RA, Greenbaum LE, Scearce LM, Kovalovich K, Taub R (1999). Liver-specific and proliferation-induced deoxyribonuclease I hypersensitive sites in the mouse insulin-like growth factor binding protein-1 gene. Hepatology.

[20] Subramaniam A, Jones WK, Gulick J, Wert S, Neumann J, Robbins J (1991). Tissue-specific regulation of the alpha-myosin heavy chain gene promoter in transgenic mice. J. Biol. Chem..

[21] Boggs JM (2006). Myelin basic protein: a multifunctional protein. Cell Mol. Life Sci..

[22] Cheng A, Li M, Liang Y, Wang Y, Wong L, Chen C, Vlassov AV, Magdaleno S (2009). Stem-loop RT-PCR quantification of siRNAs *in vitro* and *in vivo*. Oligonucleotides.

[23] Stratford S, Stec S, Jadhav V, Seitzer J, Abrams M, Beverly M (2008). Examination of real-time polymerase chain reaction methods for the detection and quantification of modified siRNA. Anal. Biochem..

[24] Raymond CK, Roberts BS, Garrett-Engele P, Lim LP, Johnson JM (2005). Simple, quantitative primer-extension PCR assay for direct monitoring of microRNAs and short-interfering RNAs. RNA.

[25] Li J, Makrigiorgos GM (2007). Anti-primer quenching-based real-time PCR for simplex or multiplex DNA quantification and single-nucleotide polymorphism genotyping. Nat. Protoc..

[26] Chen C, Ridzon DA, Broomer AJ, Zhou Z, Lee DH, Nguyen JT, Barbisin M, Xu NL, Mahuvakar VR, Andersen MR (2005). Real-time quantification of microRNAs by stem-loop RT-PCR. Nucleic Acids Res..

[27] Sempere LF, Freemantle S, Pitha-Rowe I, Moss E, Dmitrovsky E, Ambros V (2004). Expression profiling of mammalian microRNAs uncovers a subset of brain-expressed microRNAs with possible roles in murine and human neuronal differentiation. Genome Biol..

[28] Landgraf P, Rusu M, Sheridan R, Sewer A, Iovino N, Aravin A, Pfeffer S, Rice A, Kamphorst AO, Landthaler M (2007). A mammalian microRNA expression atlas based on small RNA library sequencing. Cell.

[29] Lagos-Quintana M, Rauhut R, Yalcin A, Meyer J, Lendeckel W, Tuschl T (2002). Identification of tissue-specific microRNAs from mouse. Curr. Biol..

[30] Pinheiro VB, Taylor AI, Cozens C, Abramov M, Renders M, Zhang S, Chaput JC, Wengel J, Peak-Chew SY, McLaughlin SH (2012). Synthetic genetic polymers capable of heredity and evolution. Science.

[31] Harcourt EM, Kool ET (2012). Amplified microRNA detection by templated chemistry. Nucleic Acids Res..

[32] Geary RS, Watanabe TA, Truong L, Freier S, Lesnik EA, Sioufi NB, Sasmor H, Manoharan M, Levin AA (2001). Pharmacokinetic properties of 2′-O-(2-methoxyethyl)-modified oligonucleotide analogs in rats. J. Pharmacol. Exp. Ther..

[33] Ming X (2011). Cellular delivery of siRNA and antisense oligonucleotides via receptor-mediated endocytosis. Expert Opin. Drug Deliv..

[34] Liao S, Liu Y, Zeng J, Li X, Shao N, Mao A, Wang L, Ma J, Cen H, Wang Y (2010). Aptamer-based sensitive detection of target molecules via RT-PCR signal amplification. Bioconjug. Chem..

[35] Lin JS, McNatty KP (2009). Aptamer-based regionally protected PCR for protein detection. Clin. Chem..

[36] Wang XL, Li F, Su YH, Sun X, Li XB, Schluesener HJ, Tang F, Xu SQ (2004). Ultrasensitive detection of protein using an aptamer-based exonuclease protection assay. Anal. Chem..

[37] Zhang H, Wang Z, Li XF, Le XC (2006). Ultrasensitive detection of proteins by amplification of affinity aptamers. Angew. Chem. Int. Ed. Engl..

