# Supplementary material for: Whole-body scanning PCR; a highly sensitive method to study the biodistribution of mRNAs, noncoding RNAs and therapeutic oligonucleotides
Source: Nucleic Acids Res. 2013 Jun 13;41(15):e145. doi: 10.1093/nar/gkt515 (PMC3753639; doi:10.1093/nar/gkt515)
Supplement: Supplementary Data [file supp_41_15_e145__index.html]

Whole-body scanning PCR; a highly sensitive method to study the biodistribution of mRNAs, noncoding RNAs and therapeutic oligonucleotides — Whole-body scanning PCR; a highly sensitive method to study the biodistribution of mRNAs, noncoding RNAs and therapeutic oligonucleotides — Supplementary Data 

# Whole-body scanning PCR; a highly sensitive method to study the biodistribution of mRNAs, noncoding RNAs and therapeutic oligonucleotides

## Supplementary Data

files

**Files in this Data Supplement:**

- Supplementary Data - pdf file
