# Supplementary material for: Whole-body scanning PCR; a highly sensitive method to study the biodistribution of mRNAs, noncoding RNAs and therapeutic oligonucleotides
Source: Nucleic Acids Res. 2013 Jun 13;41(15):e145. doi: 10.1093/nar/gkt515 (PMC3753639; doi:10.1093/nar/gkt515)
Supplement: Supplementary Data [file supp_gkt515_nar-00168-met-g-2013-File006.pdf]

Supplementary figures

Figure S1 | Workflow of the Whole Body Scanning PCR procedure (WBS-PCR). Spatial deconvolution of the sagittal cross-section of a mouse is achieved by placing the section on a 1536-well plate filled with lysis buffer. Lysates were transferred into 384-well plates, diluted and subjected to target quantification. Signals were convoluted using Excel spreadsheet software and converted into a TissueView imaging software compatible image file, using a macro. Biodistribution was visualized by importing and overlaying the converted Excel-data with a picture of the sagittal cross-section.

Figure S2 | Characterization of mRNAs-specific Taqman gene assays. Expression profiling using gene-specific Taqman assays detecting Insulin-like Growth Factor Binding Protein 1 (IGFBP1), Myosin heavy chain 6 (Myh6) and Myelin Basic Protein (Mbp) using total RNA purified from Thymus (Th), Lung (Lu), Heart (H), Skeletal Muscle (S.M.), Kidney (K), Brain (B), Liver (Li), Spleen (S) and Spinal cord (S.c.). Maximum averaged signal for each mRNA was set to 100%.

Figure S3 | Standard curves obtained for the quantification of let-7 miRNA family members. (**a**) Sequence alignment between members of the let-7 miRNA family (bold, underlined indicates differences in sequences compared to Let-7a). (**b**-**i**) Standard curves, obtained for the absolute quantification of let-7 miRNA family members, prepared by serial dilutions of synthetic sequences spiked into 10 ng/μL poly A.

Figure S4 | Characterization of miRNAs-specific assays. miRNAs-specific assays were run on a panel of total RNA extracted from Thymus (Th), Lung (Lu), Heart (H), Skeletal Muscle (S.M.), Kidney (K), Brain (B), Liver (Li), Spleen (S) and Spinal cord (S.c.). Maximum averaged signal for each miRNA was set to 100%.

Figure S5 | Mrp4 siRNA standard curve. Mrp4 siRNA duplex spiked and serially diluted using 10 ng/μL rat liver total RNA as diluent.

Figure S6 | Detection of various AMO-miR-16 chemical formats by RT-qPCR. AMO-miR-16 was spiked and serially diluted using 10 ng/μL. Three chemical formats were tested including RNA (**a**), 2'OMe (**b**), and MOE (**c**). Values are averages of 4 measurements. Error bars, STDEV (n=4).

Figure S7 | Molecular mechanism of the Chemical Ligation qPCR. (**a**) PS (orange) and BPS (green) ligators react with each other upon hybridization to their target sequence (blue) to form a ligated product containing a phosphorothioate linkage. During the first cycle of the qPCR-reaction, target hybridization of the reverse primer (black) will result in the formation of dsDNA. During subsequent cycles, fluorescently labeled forward primer (red) hybridize to the elongated reverse primer resulting in a fluorescent dsDNA whereas free forward primers are quenched by an anti-primer sequence (purple) labeled with a quencher resulting in an exponential increase in fluorescence signal. (**b**) AMO-miR-16 sequence variants (red, bold) were spiked and serially diluted using 10 ng/μL Poly(A) as a diluent. Detection of the targets was performed using four different BPS-ligators, where the BPS-group was coupled to an Deoxyadenosine (BPS-A), a Deoxyguanosine (BPS-G), a Thymidine (BPS-T) or a Deoxycytidine (BPS-C) (red, underlined). Values are average of 4 measurements. Error bars, STDEV (n=4).

Figure S8 | Detection of various AMO-miR-16 chemical formats by CL-qPCR. AMO-miR-16 was spiked and serially diluted using 10 ng/μL. Three chemical formats were tested including RNA (**a**), 2'OMe (**b**), and MOE (**c**). Values are averages of 4 measurements. Error bars, STDEV (n=4).

Figure S9 | AMO-miR-16 standard curve in plasma. AMO-miR-16 was spiked and serially diluted using 1:1000 diluted plasma collected from a PBS treated mouse as diluent. Values are average of 4 measurements. Error bars, STDEV (n=4).

Figure S10 | AMO-miR-16 standard curves in tissue extracts. AMO-miR-16 was spiked and serially diluted using 1:750 diluted tissue homogenates prepared from the (**a**) brain, (**b**) kidney,

(**c**) liver, (**d**) lung and (**e**) spleen of a PBS treated animal. Values are average of 4 measurements. Error bars, STDEV (n=4).

Figure S11 | miR-16 standard curve. Synthetic miR-16 RNA was spiked and serially diluted using 10 ng/μL Poly(A) as diluent. Values are average of 3 measurements. Error bars, STDEV (n=3).

Figure S12 | miR-191 standard curve. Synthetic miR-191 RNA was spiked and serially diluted using 10 ng/μL Poly(A) as diluent. Values are average of 3 measurements. Error bars, STDEV (n=3).

Figure S13 | Synthesis of 5'-BPS-coupled phosphoramidites. (**a**) Coupling of the biphenylsulfonyl to deoxynucleosides. (**b**) Generation of N-protected 5'-O-biphenylsulfonyl-2'-deoxynucleoside phosphoramidites.

Table S1 | Relative mRNA expression levels in tissue lysates. Numerical values corresponding to **Figure 1a**. Results are shown as % relative expression, where maximum averaged signal for each mRNA was set to 100%.

Table S2 | Relative mRNA expression levels in purified total RNA. Numerical values corresponding to **Supplementary Figure 2**. Results are shown as % relative expression, where maximum averaged signal for each mRNA was set to 100%.

Table S3 | Relative miRNA expression levels in tissue lysates. Numerical values corresponding to **Figure 2c**. Results are shown as % relative expression, where maximum averaged signal for each miRNA was set to 100%.

Table S4 | Relative miRNA expression levels in purified total RNA. Numerical values corresponding to **Supplementary Figure 4**. Results are shown as % relative expression, where maximum averaged signal for each miRNA was set to 100%.

Table S5 | miRNA and siRNA RT-qPCR reagents. Sequences of the forward and reverse primers used in this study, as well as their target sequence. Sequences are oriented in the 5'-3' direction and are annotated according to the 18th release of the miRBase database.

Table S6 | mRNA and rRNA probes. References of the ABI TaqMan probes used in the study.

Table S7 | NMR data obtained for the characterization of 5'BPS-coupled deoxynucleosides and 5'-O-biphenylsulfonyl-2'-deoxynucleoside phosphoramidites.

Fig.S1

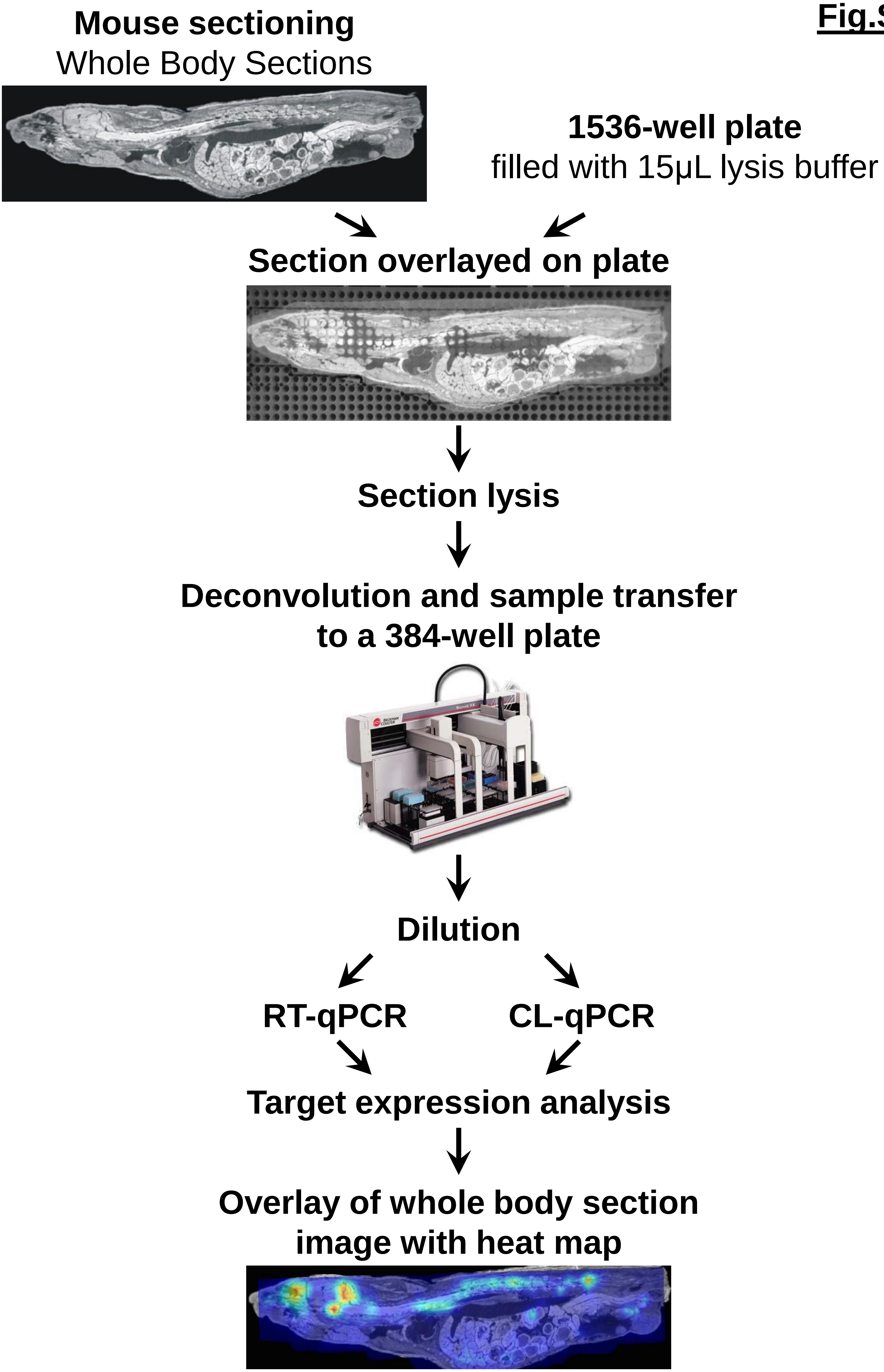
Mouse sectioning
Whole Body Sections
1536-well plate
filled with 15µL lysis buffer
Section overlayed on plate
Section lysis
Deconvolution and sample transfer
to a 384-well plate
Dilution
RT-qPCR
CL-qPCR
Target expression analysis
Overlay of whole body section
image with heat map

Fig.S2

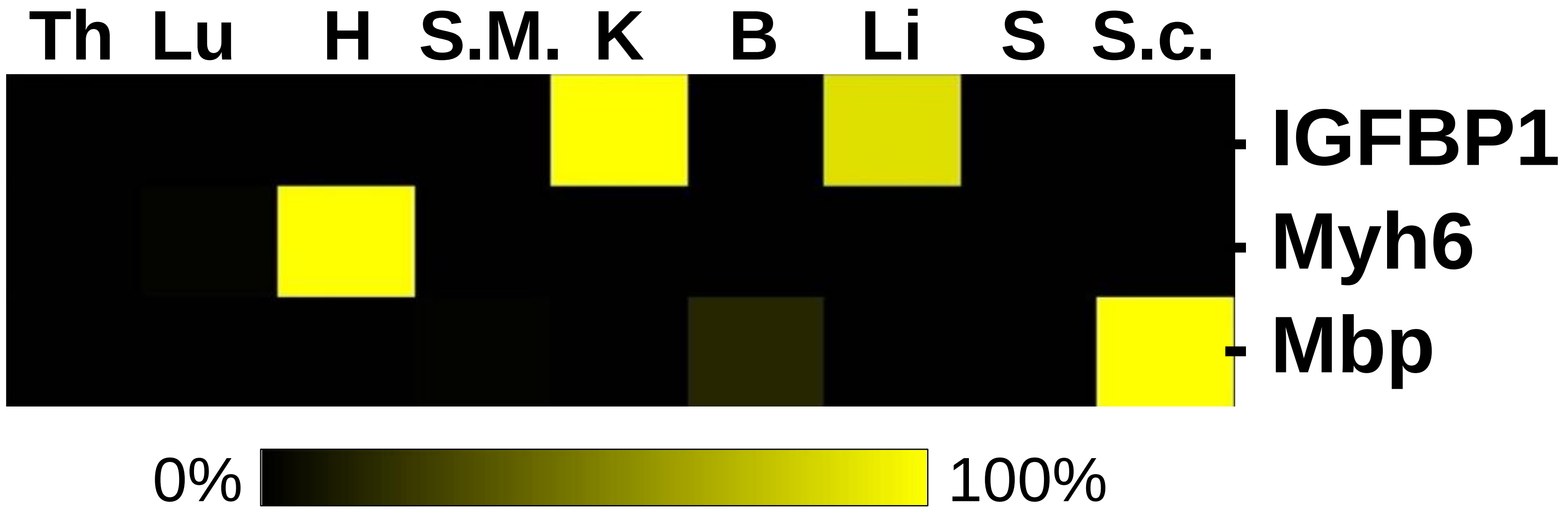

Fig.S3

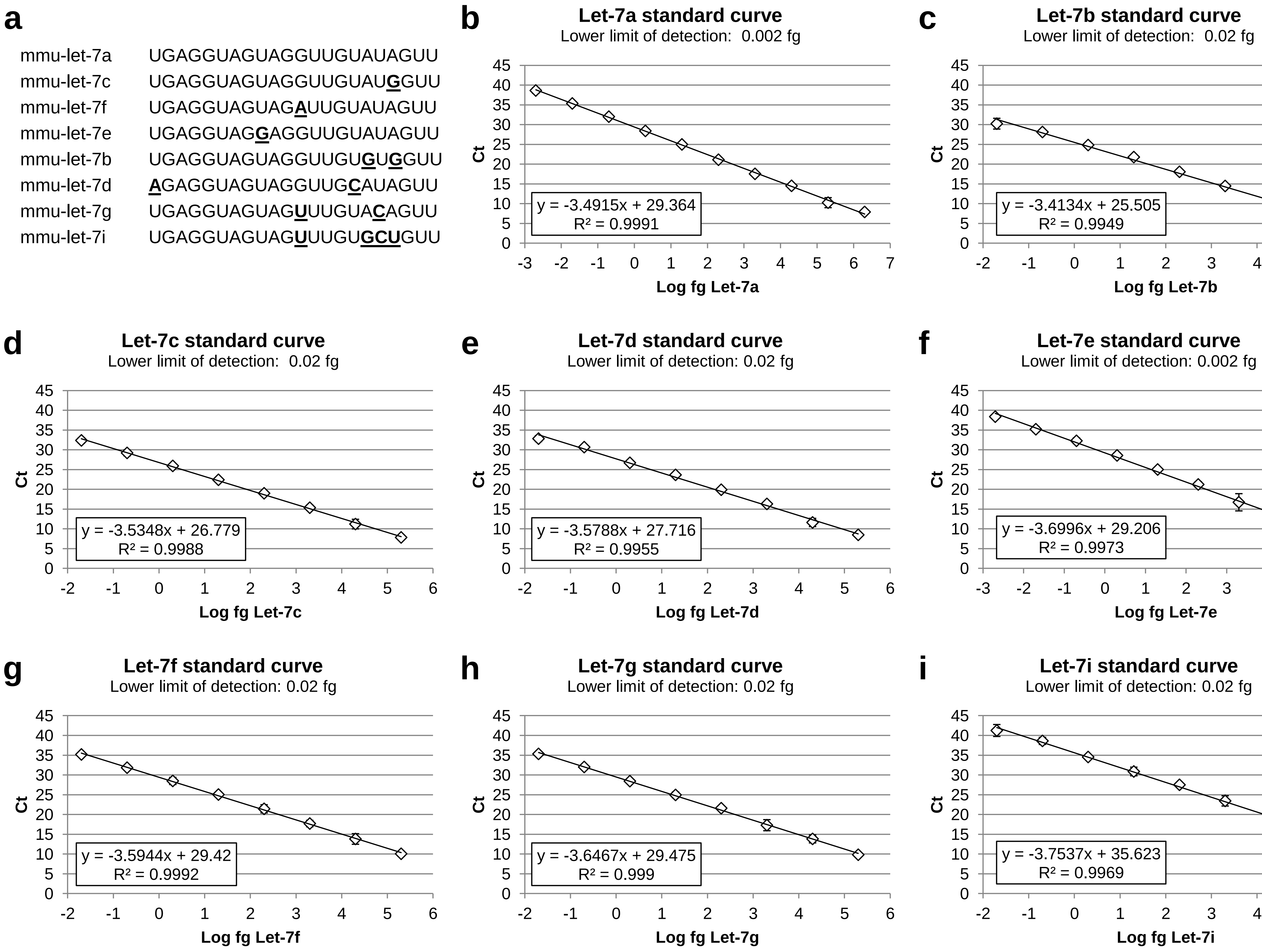
a
mmu-let-7a UGAGGUAGUAGGUUGUAUAGUU
mmu-let-7c UGAGGUAGUAGGUUGUAUGGUU
mmu-let-7f UGAGGUAGUAGAUUGUAUAGUU
mmu-let-7e UGAGGUAGGAGGUUGUAUAGUU
mmu-let-7b UGAGGUAGUAGGUUGUGUGGUU
mmu-let-7d AGAGGUAGUAGGUUGCAUAGUU
mmu-let-7g UGAGGUAGUAGUUUGUACAGUU
mmu-let-7i UGAGGUAGUAGUUUGUGCUGUU
b
Let-7a standard curve
Lower limit of detection: 0.002 fg
y = -3.4915x + 29.364
R² = 0.9991
Ct
Log fg Let-7a
c
Let-7b standard curve
Lower limit of detection: 0.02 fg
y = -3.4134x + 25.505
R² = 0.9949
Ct
Log fg Let-7b
d
Let-7c standard curve
Lower limit of detection: 0.02 fg
y = -3.5348x + 26.779
R² = 0.9988
Ct
Log fg Let-7c
e
Let-7d standard curve
Lower limit of detection: 0.02 fg
y = -3.5788x + 27.716
R² = 0.9955
Ct
Log fg Let-7d
f
Let-7e standard curve
Lower limit of detection: 0.002 fg
y = -3.6996x + 29.206
R² = 0.9973
Ct
Log fg Let-7e
g
Let-7f standard curve
Lower limit of detection: 0.02 fg
y = -3.5944x + 29.42
R² = 0.9992
Ct
Log fg Let-7f
h
Let-7g standard curve
Lower limit of detection: 0.02 fg
y = -3.6467x + 29.475
R² = 0.999
Ct
Log fg Let-7g
i
Let-7i standard curve
Lower limit of detection: 0.02 fg
y = -3.7537x + 35.623
R² = 0.9969
Ct
Log fg Let-7i

Fig.S4

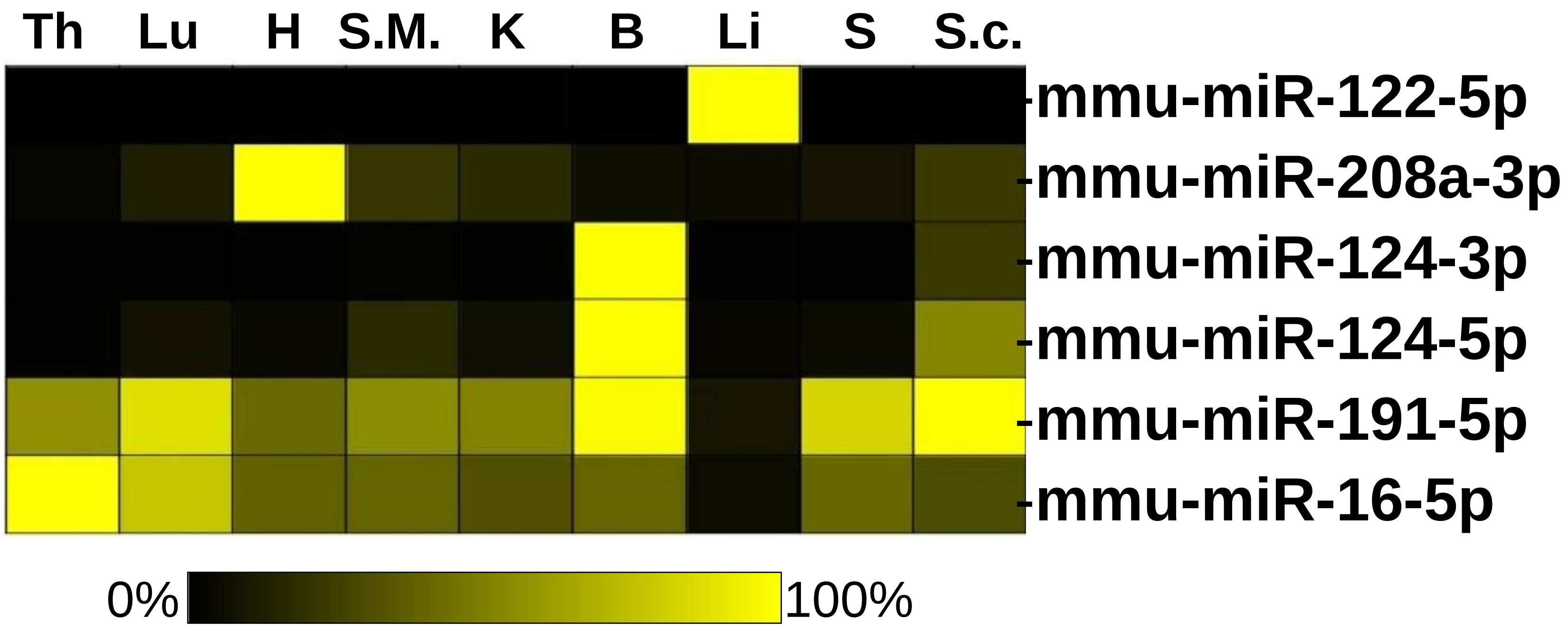

Th
Lu
H
S.M.
K
B
Li
S
S.c.
mmu-miR-122-5p
mmu-miR-208a-3p
mmu-miR-124-3p
mmu-miR-124-5p
mmu-miR-191-5p
mmu-miR-16-5p
0%
100%

Fig.S5

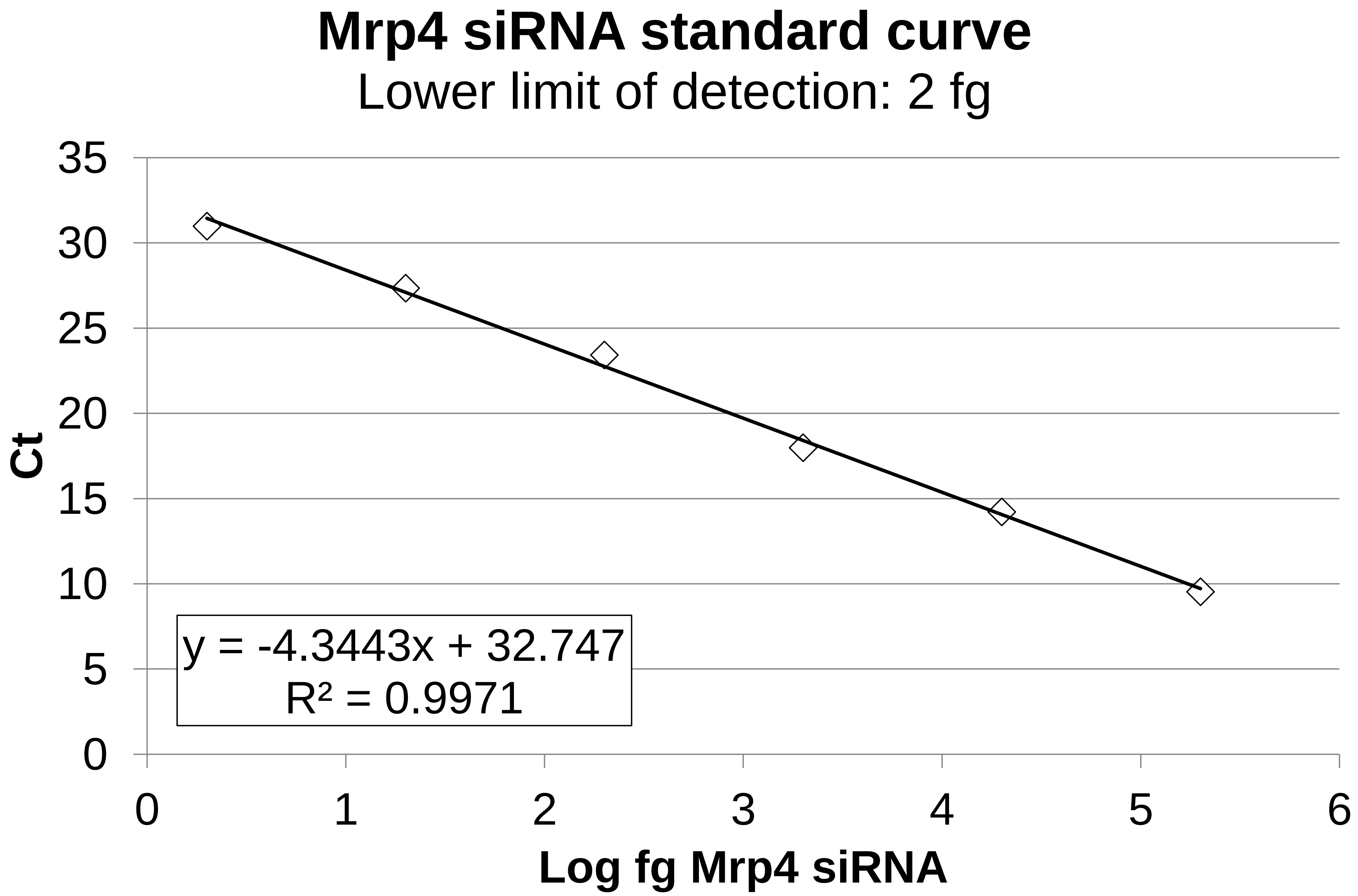
Mrp4 siRNA standard curve
Lower limit of detection: 2 fg
Ct
35
30
25
20
15
10
5
0
0
1
2
3
4
5
6
Log fg Mrp4 siRNA
y = -4.3443x + 32.747
R² = 0.9971

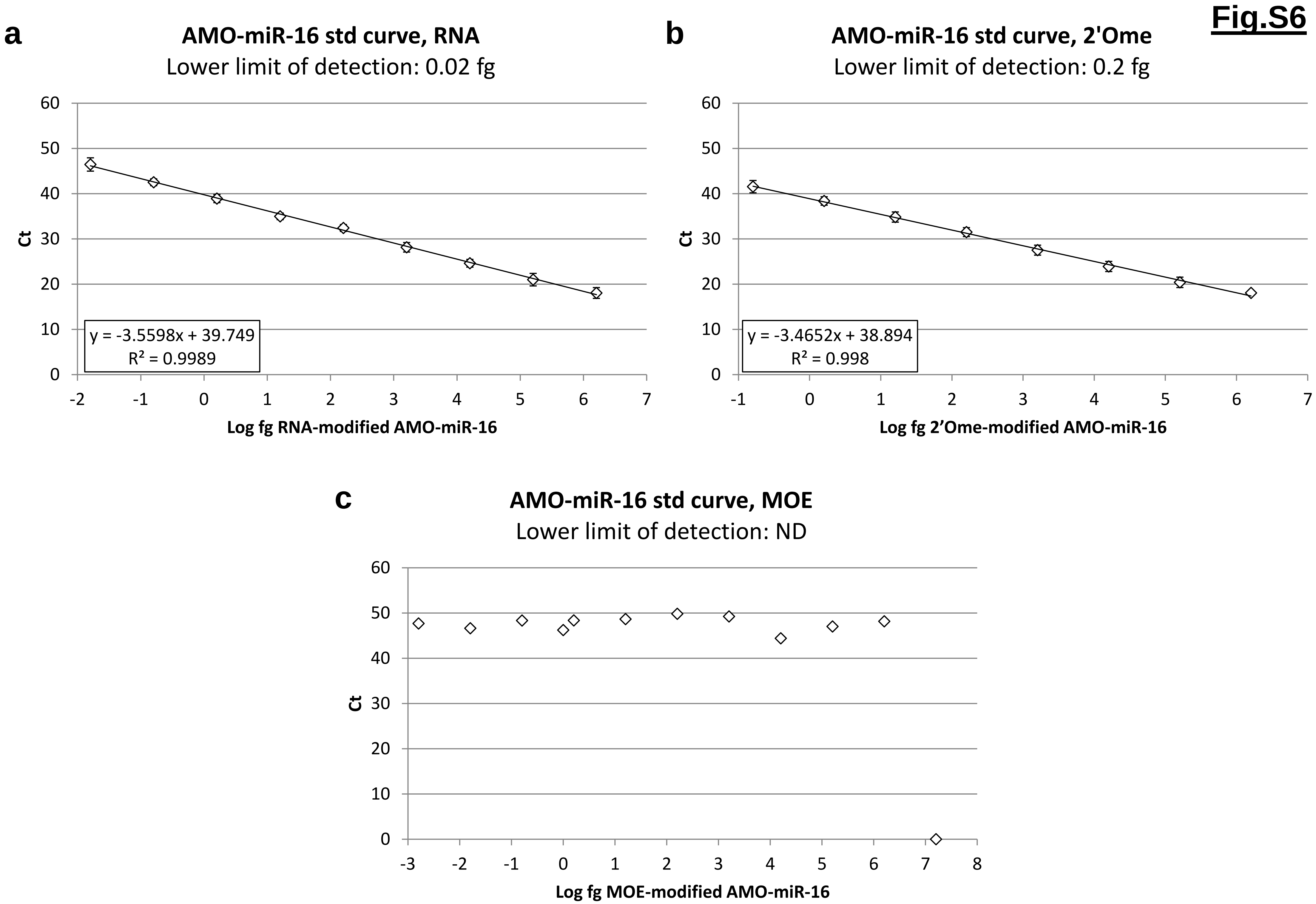
Fig.S6
a
AMO-miR-16 std curve, RNA
Lower limit of detection: 0.02 fg
Ct
y = -3.5598x + 39.749
R² = 0.9989
Log fg RNA-modified AMO-miR-16
b
AMO-miR-16 std curve, 2'Ome
Lower limit of detection: 0.2 fg
Ct
y = -3.4652x + 38.894
R² = 0.998
Log fg 2'Ome-modified AMO-miR-16
c
AMO-miR-16 std curve, MOE
Lower limit of detection: ND
Ct
Log fg MOE-modified AMO-miR-16

Fig.S7

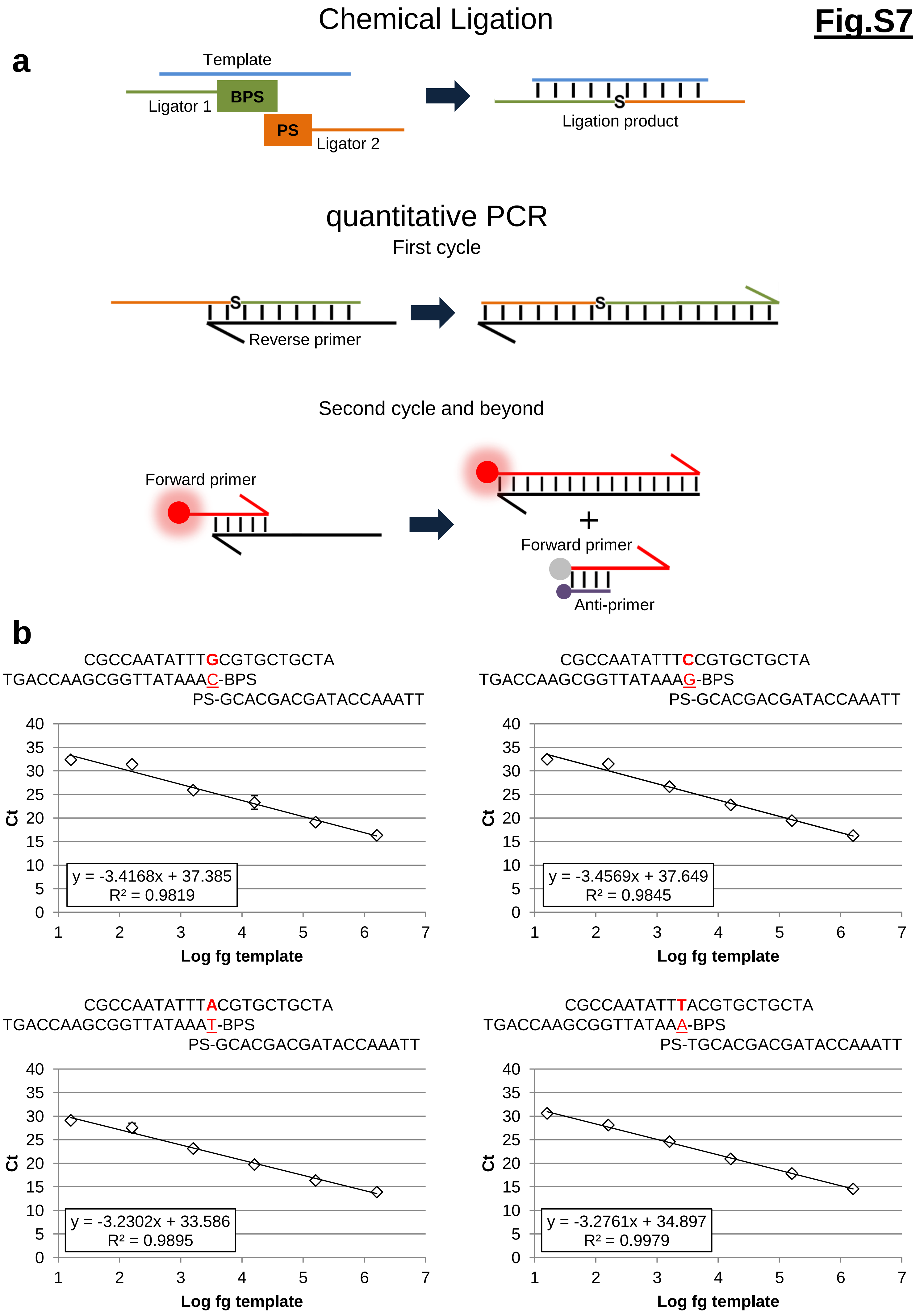
Chemical Ligation
a
Template
Ligator 1
BPS
PS
Ligator 2
S
Ligation product
quantitative PCR
First cycle
S
Reverse primer
S
Second cycle and beyond
Forward primer
+
Forward primer
Anti-primer
b
CGCCAATATTTGCGTGCTGCTA
TGACCAAGCGGTTATAAAC-BPS
PS-GCACGACGATACCAAATT
Ct
Log fg template
y = -3.4168x + 37.385
R² = 0.9819
CGCCAATATTTCCGTGCTGCTA
TGACCAAGCGGTTATAAAG-BPS
PS-GCACGACGATACCAAATT
Ct
Log fg template
y = -3.4569x + 37.649
R² = 0.9845
CGCCAATATTTACGTGCTGCTA
TGACCAAGCGGTTATAAAT-BPS
PS-GCACGACGATACCAAATT
Ct
Log fg template
y = -3.2302x + 33.586
R² = 0.9895
CGCCAATATTTACGTGCTGCTA
TGACCAAGCGGTTATAAA-BPS
PS-TGCACGACGATACCAAATT
Ct
Log fg template
y = -3.2761x + 34.897
R² = 0.9979

Fig.S8

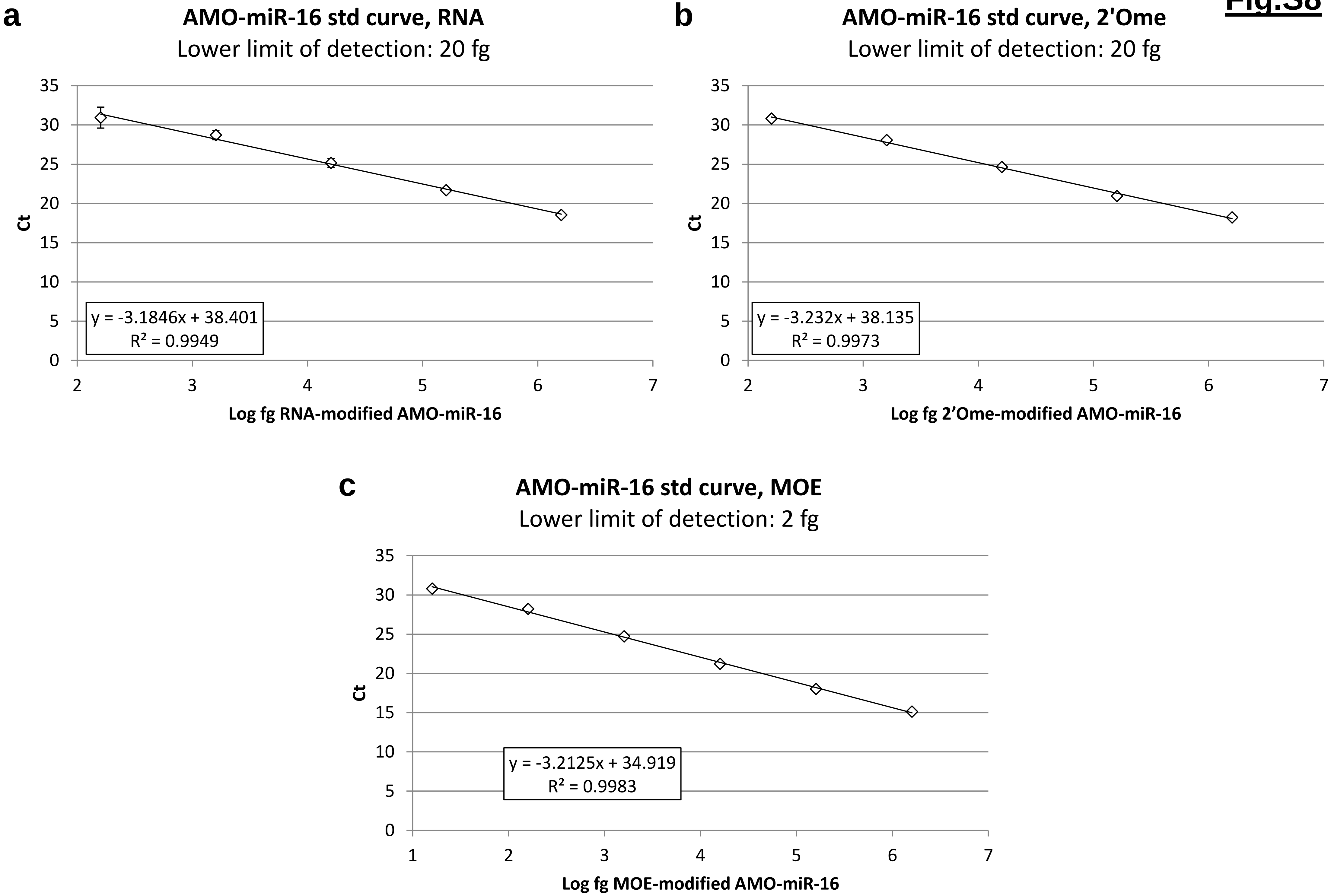
a
AMO-miR-16 std curve, RNA
Lower limit of detection: 20 fg
Ct
y = -3.1846x + 38.401
R² = 0.9949
Log fg RNA-modified AMO-miR-16
b
AMO-miR-16 std curve, 2'Ome
Lower limit of detection: 20 fg
Ct
y = -3.232x + 38.135
R² = 0.9973
Log fg 2'Ome-modified AMO-miR-16
c
AMO-miR-16 std curve, MOE
Lower limit of detection: 2 fg
Ct
y = -3.2125x + 34.919
R² = 0.9983
Log fg MOE-modified AMO-miR-16

Fig.S9

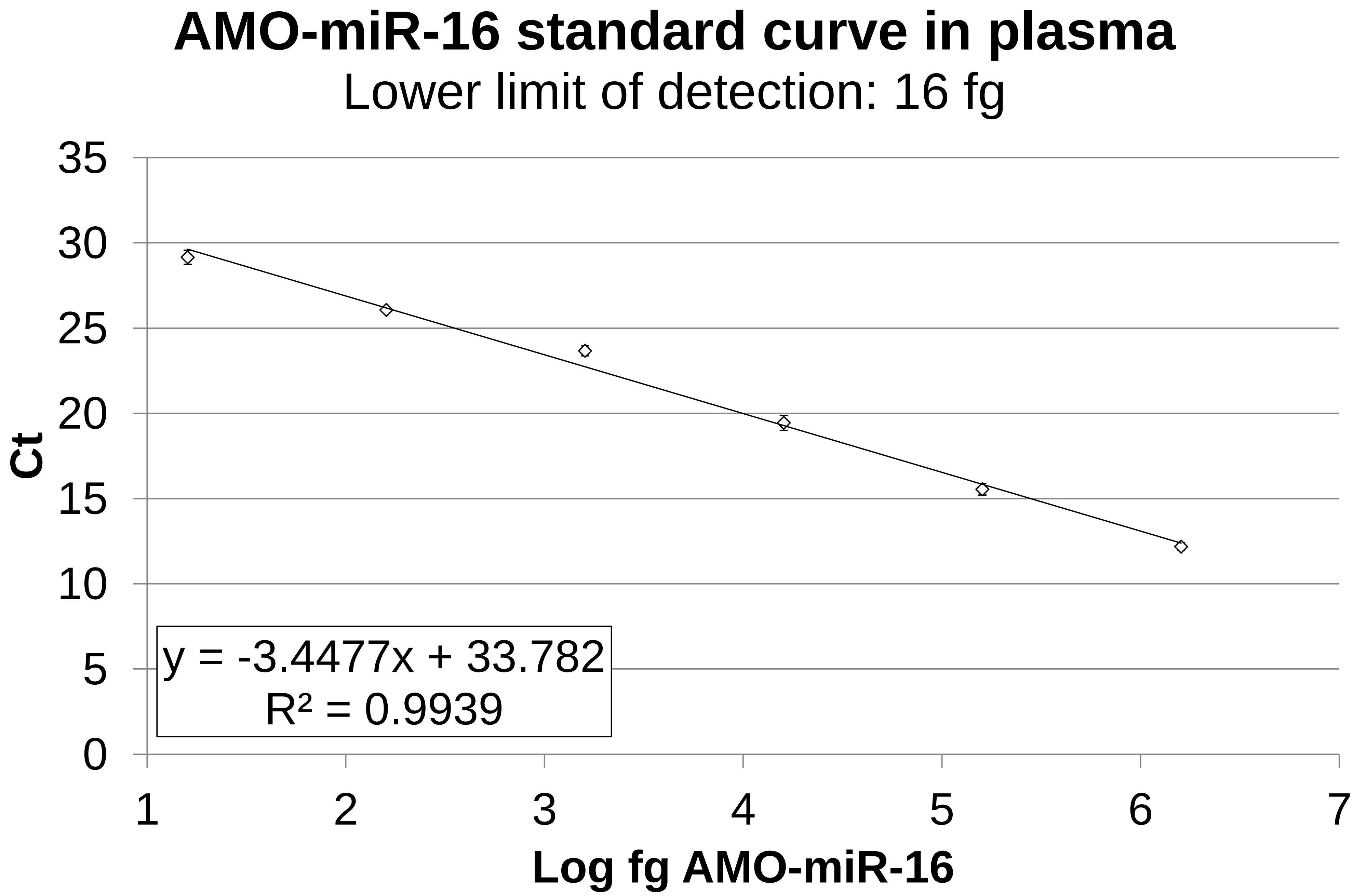
AMO-miR-16 standard curve in plasma
Lower limit of detection: 16 fg
35
30
25
20
15
10
5
0
Ct
y = -3.4477x + 33.782
R² = 0.9939
1
2
3
4
5
6
7
Log fg AMO-miR-16

Fig.S10

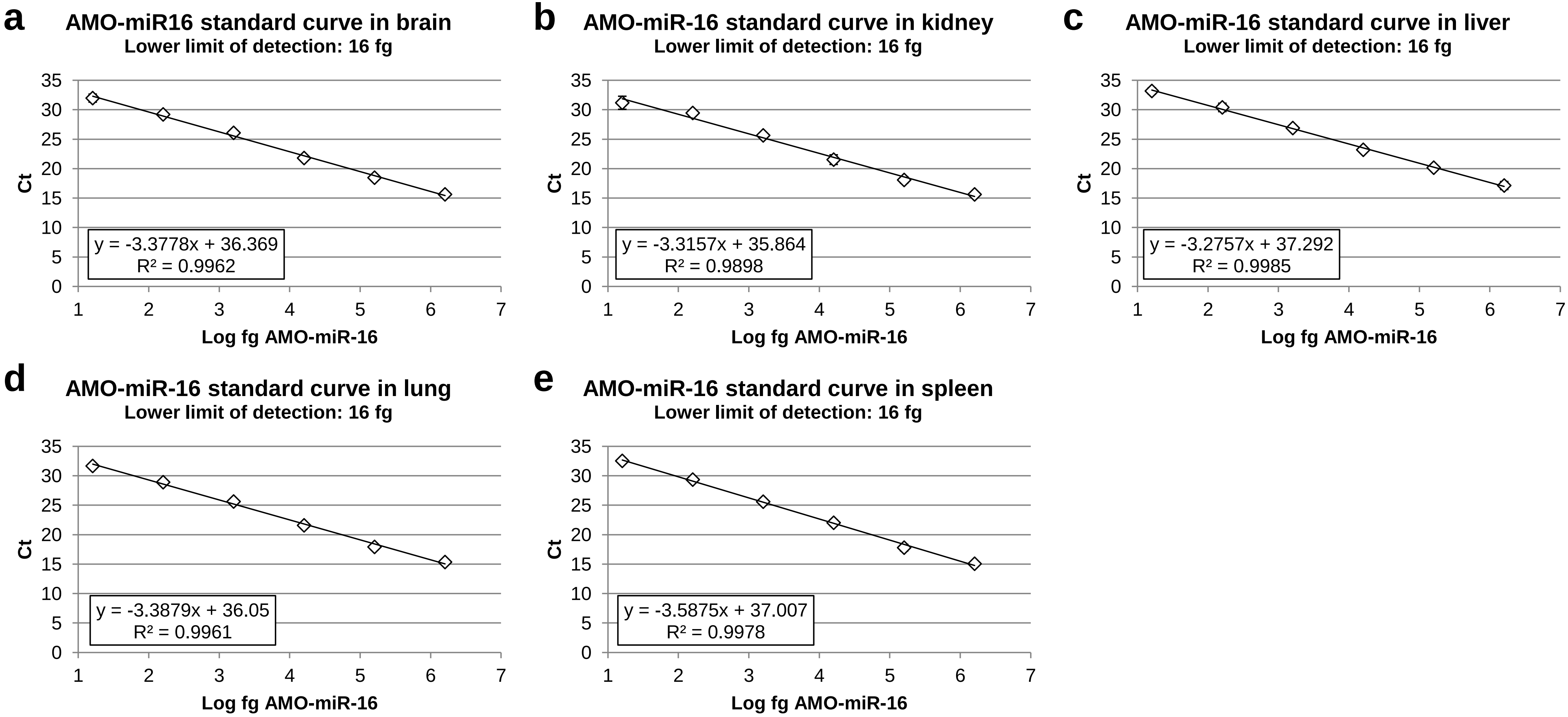
a
AMO-miR16 standard curve in brain
Lower limit of detection: 16 fg
y = -3.3778x + 36.369
R² = 0.9962
b
AMO-miR-16 standard curve in kidney
Lower limit of detection: 16 fg
y = -3.3157x + 35.864
R² = 0.9898
c
AMO-miR-16 standard curve in liver
Lower limit of detection: 16 fg
y = -3.2757x + 37.292
R² = 0.9985
d
AMO-miR-16 standard curve in lung
Lower limit of detection: 16 fg
y = -3.3879x + 36.05
R² = 0.9961
e
AMO-miR-16 standard curve in spleen
Lower limit of detection: 16 fg
y = -3.5875x + 37.007
R² = 0.9978
Ct
Log fg AMO-miR-16
0
5
10
15
20
25
30
35
1
2
3
4
5
6
7

Fig.S11

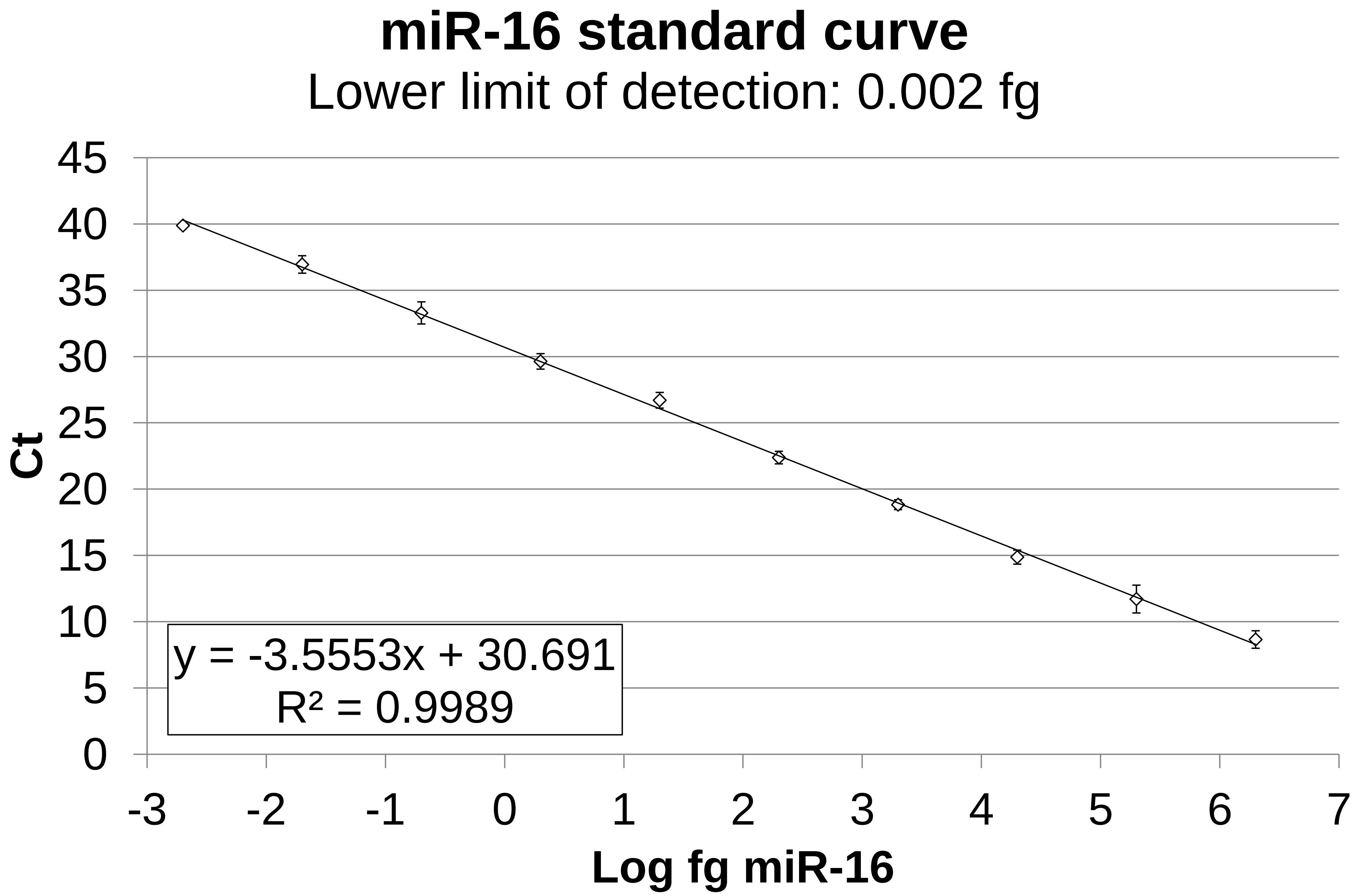
miR-16 standard curve
Lower limit of detection: 0.002 fg
Ct
45
40
35
30
25
20
15
10
5
0
-3
-2
-1
0
1
2
3
4
5
6
7
Log fg miR-16
y = -3.5553x + 30.691
R² = 0.9989

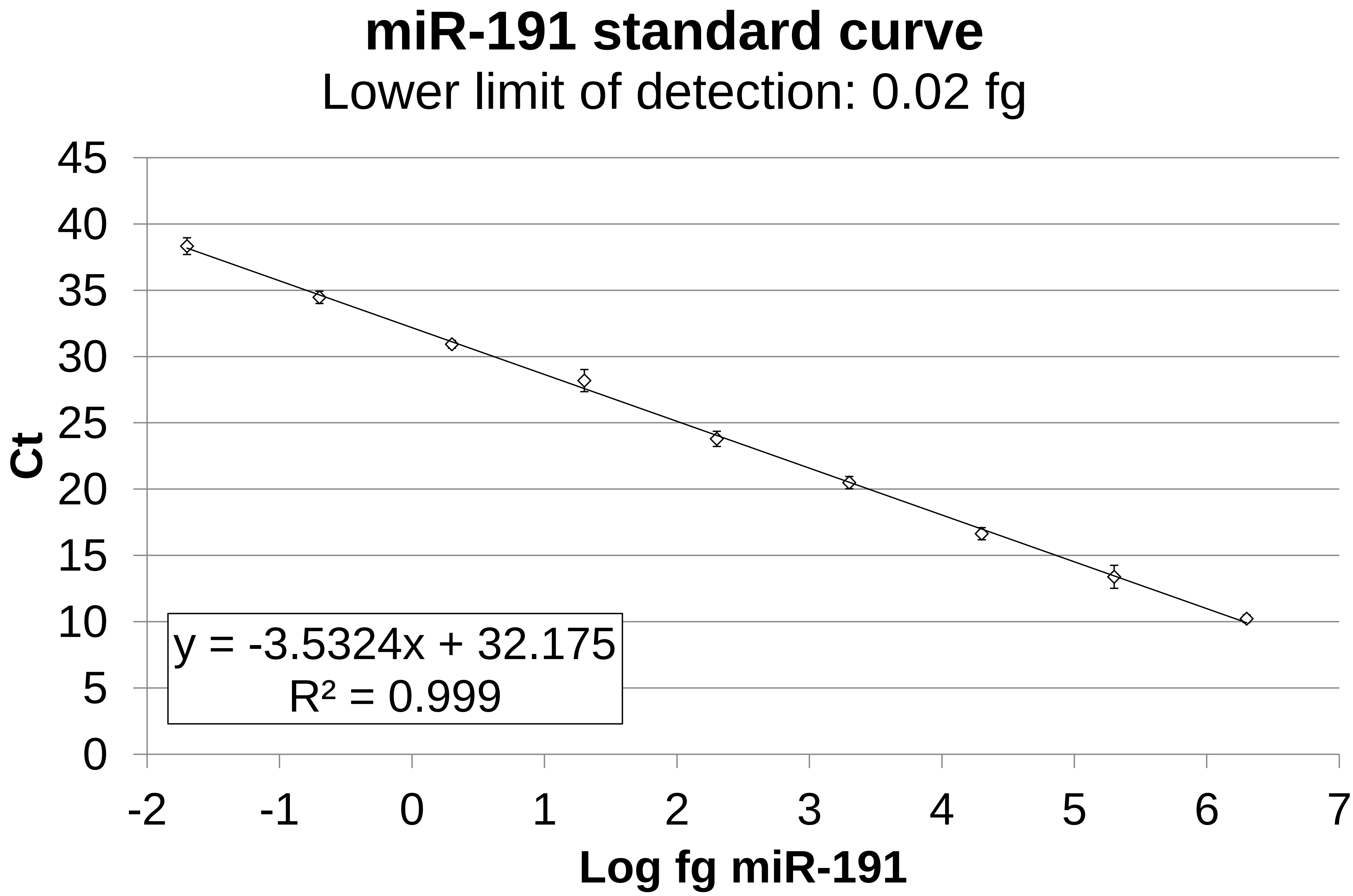
miR-191 standard curve
Lower limit of detection: 0.02 fg
Ct
45
40
35
30
25
20
15
10
5
0
-2
-1
0
1
2
3
4
5
6
7
Log fg miR-191
y = -3.5324x + 32.175
R² = 0.999

Fig.S13

a

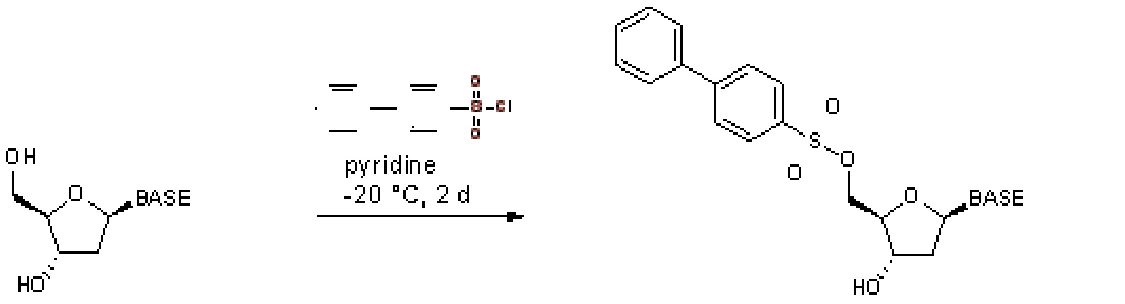

BASE = N[6]-benzoyladenine	**1**		
N[4]-benzoylcytosine	**2**		
N[2]-isobutyrylguanine	**3**		
thymine	**4**		

		Yield
BASE = N[6]-benzoyladenine	**5**	60%
N[4]-benzoylcytosine	**6**	40%
N[2]-isobutyrylguanine	**7**	50%
thymine	**8**	66%

b

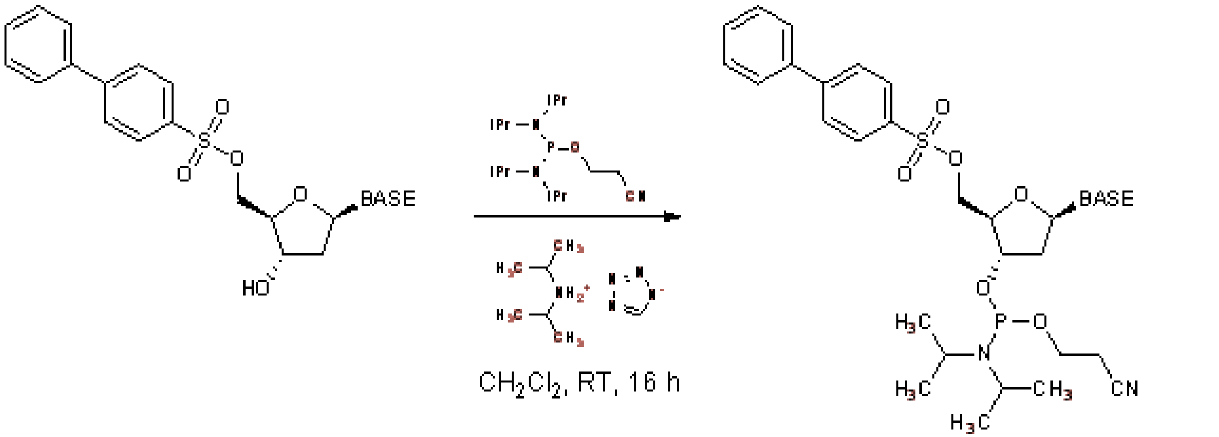

5 - 8

		Yield
BASE = N[6]-benzoyladenine	**9**	78%
N[4]-benzoylcytosine	**10**	90%
N[2]-isobutyrylguanine	**11**	85%
thymine	**12**	56%

Table.S1

	Thymus	Lung	Heart	Skeletal Muscle	Kidney	Brain	Liver	Spleen
**IGFBP1**	0.00	0.00	0.00	0.00	100.00 +/- 20.64	1.36 +/- 2.44	28.46 +/- 13.71	0.00
**Myh6**	0.00	0.10 +/- 0.03	100.00 +/- 8.43	0.00	0.00	0.00	0.00	0.00
**Mbp**	0.00	0.00	0.04 +/- 0.03	0.60 +/- 0.31	0.02 +/- 0.01	100.00 +/- 20.91	0.00	0.00

Table.S2

	Thymus	Lung	Heart	Skeletal Muscle	Kidney	Brain	Liver	Spleen	Spinal Cord
**IGFBP1**	0.00	0.00	0.00	0.00	100.0 +/- 5.21	0.00	87.86 +/- 15.93	0.00	0.00
**Myh6**	0.12 +/- 0.03	1.72 +/- 0.66	100.00 +/- 14.19	0.05 +/- 0.01	0.00	0.00	0.00	0.00	0.00
**Mbp**	0.02 +/- 0.01	0.10 +/- 0.03	0.04 +/- 0.01	1.20 +/- 0.29	0.02 +/- 0.01	14.92 +/- 1.13	0.00	0.02 +/- 0.01	100.00 +/- 13.92

Table.S3

	Thymus	Lung	Heart	Skeletal Muscle	Kidney	Brain	Liver	Spleen
mmu-miR-208a-3p	0.00	0.00	100.00 +/- 47.04	0.00	0.00	0.00	0.00	0.00
mmu-miR-1a-3p	0.09 +/- 0.08	0.06 +/- 0.05	100.00 +/- 39.91	45.51 +/- 36.57	0.00	0.06 +/- 0.10	0.00	0.00
mmu-miR-126-3p	16.57 +/- 8.54	48.04 +/- 28.38	100.00 +/- 30.78	14.08 +/- 3.69	4.22 +/- 1.26	8.28 +/- 3.88	6.18 +/- 2.85	5.03 +/- 5.44
mmu-miR-133a-5p	0.14 +/- 0.05	0.05 +/- 0.02	48.62 +/- 5.15	100.00 +/- 28.31	0.02 +/- 0.01	0.06 +/- 0.02	0.01 +/- 0.01	0.03 +/- 0.05
mmu-miR-133a-3p	0.10 +/- 0.09	0.02 +/- 0.01	40.15 +/- 9.82	100.00 +/- 24.63	0.00	0.01 +/- 0.01	0.00	0.00
mmu-miR-122-5p	0.74 +/- 0.38	0.01 +/- 0.01	0.06 +/- 0.01	0.21 +/- 0.09	0.02 +/- 0.01	0.66 +/- 0.36	100.00 +/- 35.20	0.01 +/- 0.01
mmu-miR-148b-3p	19.63 +/- 12.94	3.63 +/- 1.15	38.33 +/- 7.45	27.27 +/- 10.15	20.80 +/- 6.19	100.00 +/- 38.00	12.58 +/- 3.91	1.79 +/- 2.03
mmu-miR-139-5p	1.86 +/- 0.80	0.21 +/- 0.04	9.89 +/- 2.84	4.58 +/- 1.14	2.34 +/- 0.89	100.00 +/- 26.54	2.56 +/- 0.54	2.57 +/- 2.56
mmu-miR-139-3p	6.46 +/- 4.72	0.59 +/- 0.32	8.55 +/- 1.81	8.40 +/- 2.99	3.11 +/- 1.02	100.00 +/- 29.06	2.44 +/- 0.37	3.13 +/- 3.13
mmu-miR-124-5p	11.35 +/- 5.65	0.33 +/- 0.10	2.83 +/- 1.15	9.54 +/- 4.01	0.67 +/- 0.15	100.00 +/- 29.14	0.48 +/- 0.24	0.53 +/- 0.64
mmu-miR-137-3p	1.25 +/- 1.46	0.02 +/- 0.04	0.15 +/- 0.28	1.23 +/- 0.74	0.03 +/- 0.05	100.00 +/- 34.65	0.18 +/- 0.06	0.10 +/- 0.13
mmu-miR-127-3p	0.85 +/- 0.55	0.11 +/- 0.04	0.89 +/- 0.16	1.14 +/- 0.34	0.01 +/- 0.01	100.00 +/- 28.56	0.10 +/- 0.04	0.45 +/- 0.45
mmu-miR-132-3p	1.35 +/- 1.34	0.05 +/- 0.03	0.58 +/- 0.11	0.37 +/- 0.08	0.08 +/- 0.02	100.00 +/- 25.54	0.06 +/- 0.01	0.04 +/- 0.04
mmu-miR-128-3p	8.38 +/- 6.35	0.01 +/- 0.01	0.52 +/- 0.18	1.21 +/- 0.16	0.02 +/- 0.01	100.00 +/- 41.58	0.02 +/- 0.01	0.03 +/- 0.03
mmu-miR-124-3p	0.05 +/- 0.04	0.00	0.07 +/- 0.03	0.29 +/- 0.16	0.01 +/- 0.01	100.00 +/- 55.57	0.01 +/- 0.01	0.00
mmu-miR-125a-5P	10.87 +/- 6.93	12.83 +/- 2.19	19.93 +/- 2.87	8.57 +/- 4.28	11.11 +/- 1.72	100.00 +/- 36.21	1.36 +/- 0.31	3.02 +/- 3.30
mmu-miR-125b-1-5p	27.67 +/- 15.51	10.10 +/- 4.31	25.48 +/- 4.32	4.18 +/- 0.52	2.92 +/- 0.76	100.00 +/- 28.01	0.35 +/- 0.63	0.81 +/- 1.40
mmu-miR-125a-3P	100.00 +/- 108.61	4.69 +/- 1.35	8.30 +/- 3.66	12.78 +/- 4.44	4.38 +/- 1.17	25.21 +/- 13.21	1.76 +/- 1.34	6.76 +/- 8.41
mmu-miR-125b-3p	100.00 +/- 43.20	3.00 +/- 1.85	6.17 +/- 0.91	5.84 +/- 2.08	2.03 +/- 0.67	48.65 +/- 15.20	2.22 +/- 0.84	7.04 +/- 7.23
mmu-miR-125b-2-3p	100.00 +/- 63.41	5.46 +/- 2.35	34.83 +/- 5.43	19.48 +/- 8.22	4.85 +/- 1.54	69.08 +/- 40.36	2.46 +/- 1.56	4.49 +/- 5.67
mmu-miR-15a-5p	100.00 +/- 72.72	2.71 +/- 1.05	7.07 +/- 4.08	5.36 +/- 3.54	2.85 +/- 2.16	5.52 +/- 2.20	2.38 +/- 1.11	4.97 +/- 5.78
mmu-miR-16-5p	100.00 +/- 52.23	2.70 +/- 0.48	7.97 +/- 2.27	5.03 +/- 1.45	2.36 +/- 0.67	10.23 +/- 5.21	2.41 +/- 0.87	4.64 +/- 4.67
mmu-miR-15b-5p	100.00 +/- 55.55	1.91 +/- 0.78	5.69 +/- 0.98	1.91 +/- 0.47	1.57 +/- 0.26	1.75 +/- 0.69	1.39 +/- 0.73	5.62 +/- 7.25
mmu-miR-191-5p	100.00 +/- 43.55	3.53 +/- 0.91	33.34 +/- 4.47	17.71 +/- 7.43	15.42 +/- 6.96	43.89 +/- 10.91	5.54 +/- 0.54	22.40 +/- 22.47

Table.S4

	Thymus	Lung	Heart	Skeletal Muscle	Kidney	Brain	Liver	Spleen	Spinal Cord
**miR-122**	0.00	0.01 +/- 0.01	0.02 +/ 0.01	0.00	0.28 +/- 0.07	0.01 +/- 0.01	100.00 +/- 23.30	0.01 +/- 0.01	0.02 +/- 0.01
**miR-208a**	0.00	7.91 +/- 14.53	100.00 +/- 70.18	0.00	13.24 +/- 13.73	0.00	0.00	0.00	13.08 +/- 23.63
**miR-124-3p**	0.70 +/- 0.50	0.91 +/- 0.87	1.10 +/- 0.41	1.46 +/- 0.92	1.01 +/- 0.55	100.00 +/- 39.22	0.92 +/- 0.67	0.86 +/- 0.37	22.47 +/- 11.04
**miR-124-5p**	0.00	5.44 +/- 6.69	0.00	0.00	4.24 +/- 4.40	100.00 +/- 23.08	0.00	0.00	52.40 +/- 20.19
**miR-191**	56.51 +/- 13.38	87.43 +/- 42.12	41.16 +/- 8.21	54.99 +/- 15.42	51.06 +/- 8.12	98.00 +/- 13.10	8.43 +/- 2.68	83.26 +/- 12.55	100.00 +/- 15.63
**miR-16**	100.00 +/- 21.48	77.50 +/- 48.70	37.95 +/- 8.94	39.58 +/- 7.33	31.32 +/- 4.71	39.37 +/- 5.72	5.22 +/- 0.83	40.91 +/- 5.61	30.11 +/- 4.05

Table.S5

Sequence ID	Sequence	Forward primer	Reverse primer
mmu-miR-1a-3p	UGGAAUGUAAAGAAGUAUGUAU	FAM-CTCCCTCCCTCGATTTTGGAATGTAAAGAA	GCGCTGGATAATACATAC
mmu-miR-122-5p	UGGAGUGUGACAAUGGUGUUUG	FAM-CTCCCTCCCTCGATTTTGGAGTGTGACAAT	GCGCTGGATACAAACACC
mmu-miR-124-3p	UAAGGCACGCGGUGAAUGCC	FAM-CTCCCTCCCTCGATTTTAAGGCACGCGGT	GCGCTGGATAGGCATTC
mmu-miR-124-5p	CGUGUUCACAGCGGACCUUGAU	FAM-CTCCCTCCCTCGATTTCGTGTTCACAGCGG	GCGCTGGATAATCAAGGT
mmu-miR-125a-3P	ACAGGUGAGGUUCUUGGGAGCC	FAM-CTCCCTCCCTCGATTTACAGGTGAGGTTCT	GCGCTGGATAGGCTCCCA
mmu-miR-125a-5P	UCCCUGAGACCCUUUAACCUGUGA	FAM-CTCCCTCCCTCGATTTTCCCTGAGACCCTT	GCGCTGGATACACAGGTTA
mmu-miR-125b-2-3p	ACAAGUCAGGUUCUUGGGACCU	FAM-CTCCCTCCCTCGATTTACAAGTCAGGTTCT	GCGCTGGATAAGGTCCCA
mmu-miR-125b-1-3P	ACGGGUUAGGCUCUUGGGAGCU	FAM-CTCCCTCCCTCGATTTACGGGTTAGGCTCT	GCGCTGGATAAGCTCCCA
mmu-miR-125b-5P	UCCCUGAGACCCUAACUUGUGA	FAM-CTCCCTCCCTCGATTTTCCCTGAGACCCTA	GCGCTGGATATCACAAGT
mmu-miR-126-3P	UCGUACCGUGAGUAAUAAUGCG	FAM-CTCCCTCCCTCGATTTTCGTACCGTGAGTA	GCGCTGGATACGCATTAT
mmu-miR-126-5P	CAUUAUUACUUUUGGUACGCG	FAM-CTCCCTCCCTCGATTTCATTATTACTTTTG	GCGCTGGATACGCGTAC
mmu-miR-127-3p	UCGGAUCCGUCUGAGCUUGGCU	FAM-CTCCCTCCCTCGATTTTCGGATCCGTCTGA	GCGCTGGATAAGCCAAGC
mmu-miR-128-3p	UCACAGUGAACCGGUCUCUUU	FAM-CTCCCTCCCTCGATTTTCACAGTAACCGG	GCGCTGGATAAAAGAGA
mmu-miR-132-3p	UAACAGUCUACAGCCAUGGUCG	FAM-CTCCCTCCCTCGATTTTAACAGTCTACAGC	GCGCTGGATACGACCATG
mmu-miR-133a-3p	UUUGGUCCCCUUCAACCAGCUG	FAM-CTCCCTCCCTCGATTTTTTGGTCCCCTTCA	GCGCTGGATACAGCTGGT
mmu-miR-137-3p	UUAUUGCUUAAGAAUACGCGUAG	FAM-CTCCCTCCCTCGATTTTTATTGCTTAAGAA	GCGCTGGATACTACGCGTA
mmu-miR-139-3P	UGGAGACGCGGCCCUGUUGGAG	FAM-CTCCCTCCCTCGATTTTGGAGACGCGGCCC	GCGCTGGATACTCCAACA
mmu-miR-139-5P	UCUACAGUGCACGUGUCUCCAG	FAM-CTCCCTCCCTCGATTTTCTACAGTGCACG	GCGCTGGATACTGGAGAC
mmu-miR-148b-3p	UCAGUGCAUCACAGAACUUUGU	FAM-CTCCCTCCCTCGATTTTCAGTGCATCACAG	GCGCTGGATAACAAAGTT
mmu-miR-15a-5p	UAGCAGCACAUAAUGGUUUGUG	FAM-CTCCCTCCCTCGATTTTAGCAGCACATAAT	GCGCTGGATACACAAACC
mmu-miR-15b-5p	UAGCAGCACAUCAUGGUUUACA	FAM-CTCCCTCCCTCGATTTTAGCAGCACATCAT	GCGCTGGATATGTAAACC
mmu-miR-16-5p	UAGCAGCACGUAAAUAUUGGCG	ROX-CTCCCTCCCTCGATTTTAGCAGCACGTAAA	GATTTGTTCTGGTTCGCCAAT
mmu-miR-191-5p	CAACGGAAUCCCAAAAGCAGCUG	TAMRA-CTCCCTCCCTCGATTTCAACGGAATCCCA	GCGCTGGATAAGCTGCTTT
mmu-miR-208a-3p	AUAAGACGAGCAAAAAGCUUGU	FAM-CTCCCTCCCTCGATTTATAAGACGAGCAAA	CCGAGGTACAAGCTTTTTG
mmu-let-7a-5p	UGAGGUAGUAGGUUGUAUAGUU	FAM-CTCCCTCCCTCGATTTTGAGGTAGTAGGTT	GCGCTAAGGATAAACTAT
mmu-let-7b-5p	UGAGGUAGUAGGUUGUGUGGUU	FAM-CTCCCTCCCTCGATTTTGAGGTAGTAGGTT	GCGCTGGATAAACCACAC
mmu-let-7c-5p	UGAGGUAGUAGGUUGUAUGGUU	FAM-CTCCCTCCCTCGATTTTGAGGTAGTAGGTT	GCGCTAAGGATAAACCAT
mmu-let-7d-5p	AGAGGUAGUAGGUUGCAUAGUU	FAM-CTCCCTCCCTCGATTTAGAGGTAGTAGGTT	GCGCTGGATAAACTATGC
mmu-let-7e-5p	UGAGGUAGGAGGUUGUAUAGUU	FAM-CTCCCTCCCTCGATTTTGAGGTAGGAG	GCGCTGGATAAACTATAC
mmu-let-7f-5p	UGAGGUAGUAGAUUGUAUAGUU	FAM-CTCCCTCCCTCGATTTTGAGGTAGTAGATT	GCGCTGGATAAACTATAC
mmu-let-7g-5p	UGAGGUAGUAGUUUGUACAGUU	FAM-CTCCCTCCCTCGATTTTGAGGTAGTAGTTT	GCGCTGGATAAACTGTAC
mmu-let-7i-5p	UGAGGUAGUAGUUUGUGCUGUU	FAM-CTCCCTCCCTCGATTTTGAGGTAGTAGTTT	GCGCTGGATAAACAGCAC
Mrp4 siRNA	ACAGCUCCUGACACCUCUCdTdT	FAM-ACTCCCTCCCTCGATTTACAGCTCCTGACAC	CAAGCAGAAGACGAAGAGAGG
Chemical Ligation product AMO-miR-16	TTAAACCATAGCAGCACGTAAATAT TGGCGAACCAGT	FAM-CTCCCTCCCTCGATTTAAACCATAGCAGCACG	TCTGGTTCGCCAATATTTACG

Table.S6

Target	AoD ID
Mouse IGFBP1	Mm008334477_m1 Igfbp1
Mouse Myh6	Mm00440354_m1 Myh6
Mouse Mbp	Mm01266402_m1 Mbp
Human ELAVL1	Hs00171309_m1 ELAVL1
Human Actb	Mm006707939_s1 Actb
Human GAPDH	Mm99999915_g1 Gapdh
Genomic 18S	Hs03003631_g1 18S
18S rRNA	Hs99999901_s1 18S

Table.S7

Compound	Yield	Analytical Data
**5**	60	Solvent system for flash chromatography: dichloromethane/methanol 96:4. TLC (dichloromethane/methanol 96:4): R_f = 0.07. ^{1}H NMR (400 MHz, d_6-DMSO) δ 11.17 (s, 1H), 8.68 (s, 1H), 8.57 (s, 1H), 8.08 − 8.03 (m, 2H), 7.88 − 7.83 (m, 4H), 7.74 − 7.70 (m, 2H), 7.68 − 7.64 (m, 1H), 7.59 − 7.54 (m, 2H), 7.51 − 7.41 (m, 3H), 6.47 (t, 1H, J = 6.7 Hz), 5.59 (s br., 1H), 4.55 − 4.50 (m br., 1H), 4.40 (dd, 1H, J_1 = 10.9 Hz, J_2 = 4.1 Hz), 4.35 (dd, 1H, J_1 = 11.0 Hz, J_2 = 6.5 Hz), 4.09 − 4.05 (m, 1H), 2.90 (p, 1H, J = 6.7 Hz), 2.40 (ddd, 1H, J_1 = 13.5 Hz, J_2 = 6.7 Hz, J_3 = 4.2 Hz).
**6**	40%	Solvent system for flash chromatography: dichloromethane/methanol 96:4. TLC (dichloromethane/methanol 96:4): R_f = 0.13. ^{1}H NMR (400 MHz, $CDCl_3$) δ 9.76 (s br., 1H), 7.96 (d, 1H, J = 7.6 Hz), 7.93 − 7.90 (m, 2H), 7.82 (d, 2H, J = 7.6 Hz), 7.75 − 7.71 (m, 2H), 7.57 − 7.52 (m, 3H), 7.46 − 7.31 (m, 6H), 6.22 (t, 1H, J = 6.3 Hz), 4.47 − 4.43 (m, 1H), 4.37 (dd, 1H, J_1 = 11.2 Hz, J_2 = 2.9 Hz), 4.33 (dd, 1H, J_1 = 11.4 Hz, J_2 = 3.1 Hz), 4.20 − 4.18 (m, 1H), 3.32 (s br., 1H), 2.65 (ddd, 1H, J_1 = 13.9 Hz, J_2 = 6.0 Hz, J_3 = 4.3 Hz), 2.14 (p, 1H, J = 6.7 Hz).
**7**	50%	Solvent system for flash chromatography: dichloromethane/acetone 4:6 to 1:9. TLC (dichloromethane/acetone 4:6): R_f = 0.23. ^{1}H NMR (400 MHz, $CDCl_3$) δ 12.20 (s, 1H), 8.89 (s, 1H), 7.81 − 7.77 (m, 3H), 7.63 − 7.60 (m, 2H), 7.49 − 7.46 (m, 2H), 7.40 − 7.31 (m, 3H), 6.13 (t, 1H, J = 6.7 Hz), 4.79 − 4.76 (m, 1H), 4.32 (dd, 1H, J_1 = 10.5 Hz, J_2 = 3.2 Hz), 4.22 (dd, 1H, J_1 = 10.6 Hz, J_2 = 4.1 Hz), 4.18 − 4.15 (m, 1H), 2.90 (p, 1H, J = 6.7 Hz), 2.73 (h, 1H, J = 6.9 Hz), 2.37 (ddd, 1H, J_1 = 13.2 Hz, J_2 = 6.1 Hz, J_3 = 3.0 Hz), 1.16 (d, 3H, J = 6.8 Hz), 1.15 (d, 3H, J = 6.8 Hz). ^{13}C NMR (101 MHz, $CDCl_3$) δ 180.24 (s), 155.90 (s), 148.56 (s), 147.99 (s), 147.01 (s), 139.04 (s), 138.54 (d), 133.40 (d), 129.12 (d), 128.87 (d), 128.32 (d), 127.90 (d), 127.27 (d), 121.44 (s), 85.47 (d), 84.56 (d), 71.35 (d), 70.10 (t), 39.27 (t), 36.12 (d), 19.01 (q), 18.99 (q).
**8**	66%	Solvent system for flash chromatography: dichloromethane/methanol 96:4. TLC (dichloromethane/methanol 9:1): R_f = 0.18. ^{1}H NMR (400 MHz, d_6-DMSO) δ 11.31 (s, 1H), 8.01 − 7.96 (m, 4H), 7.78 − 7.75 (m, 2H), 7.56 − 7.37 (m, 4H), 6.17 (t, 1H, J = 6.8 Hz), 5.46 (d, 1H, J = 4.3 Hz), 4.34 (dd, 1H, J_1 = 10.8 Hz, J_2 = 3.3 Hz), 4.27 (dd, 1H, J_1 = 10.9 Hz, J_2 = 5.8 Hz), 4.23 − 4.18 (m, 1H), 3.93 − 3.90 (m, 1H), 2.18 (p, 1H, J = 6.9 Hz), 2.39 (ddd, 1H, J_1 = 13.5 Hz, J_2 = 6.5 Hz, J_3 = 2.9 Hz). ^{13}C NMR (101 MHz, d_6-DMSO) δ 163.60 (s), 150.34 (s), 145.82 (s), 138.01 (s), 135.84 (d), 133.68 (s), 129.18 (d), 128.87 (d), 128.24 (d), 127.85 (d), 127.15 (d), 109.79 (s), 84.04 (d), 83.21 (d), 70.31 (t), 69.91 (d), 38.38 (t), 12.02 (q).
**9**	78%	Solvent system for flash chromatography: heptane/ethyl acetate 1:4 + 1% triethylamine. TLC (heptane/ethyl acetate 1:4): R_f = 0.23, 0.14. ^{1}H NMR (400 MHz, $CDCl_3$) δ 8.86 (s br., 1H), 8.66 (s, 1H), 8.11, 8.08 (2s, 1H), 7.92 − 7.89 (m, 2H), 7.86 − 7.80 (m, 2H), 7.63 − 7.27 (m, 10H), 6.40 − 6.36 (m, 1H), 4.82 − 4.74 (m, 1H), 4.37 − 4.24 (m, 3H), 3.87 − 3.78 (m, 1H), 3.74 − 3.66 (m, 1H), 3.61 − 3.36 (m, 2H), 2.98 − 2.87 (m, 1H), 2.65 − 2.51 (m, 3H), 1.24 − 1.10 (m, 12H). ^{13}C NMR (101 MHz, $CDCl_3$) δ 164.57 (s), 152.57 (d), 151.29 (s), 149.60 (s), 146.94 (s), 146.92 (s), 141.78 (d), 141.66 (d), 138.74 (s), 138.71 (s), 133.79 (s), 133.61 (s), 132.78 (d), 132.59 (d), 129.11 (d), 128.84 (d), 128.81 (d), 128.42 (d), 128.39 (d), 127.89 (d), 127.81 (d), 127.77 (d), 127.37 (d), 123.49 (s), 123.46 (s), 117.77 (s), 117.62 (s), 84.96 (d), 84.83 (d), 83.96 (d, J_{CP} = 2.9 Hz), 83.68 (d, J_{CP} = 6.6 Hz), 73.62 (d, J_{CP} = 16.1 Hz), 73.33 (d, J_{CP} = 16.1 Hz), 69.17 (t), 68.99 (t), 58.33 (t), 58.15 (t), 45.63 (d, J_{CP} = 5.1 Hz), 43.36 (d, J_{CP} = 12.4 Hz), 39.49 (t, J_{CP} = 5.2 Hz), 38.73 (t, J_{CP} = 3.7 Hz), (d), 24.67 (q), 24.61 (q), 24.54 (q), 20.46 (d, J_{CP} = 7.3 Hz), 20.41 (d, J_{CP} = 7.3 Hz).
**10**	90%	Solvent system for flash chromatography: heptane/ethyl acetate 1:4 + 1% triethylamine. TLC (heptane/ethyl acetate 14:86): R_f = 0.48, 0.39. ^{1}H NMR (400 MHz, $CDCl_3$) δ 8.56 (s br., 1H), 7.95 − 7.87 (m, 3H), 7.82 − 7.78 (m, 2H), 7.76 − 7.71 (m, 2H), 7.57 − 7.52 (m, 3H), 7.46 − 7.30 (m, 6H), 6.21 − 6.17 (m, 1H), 4.52 − 4.44 (m, 1H), 4.40 − 4.22 (m, 3H), 3.87 − 3.37 (m, 4H), 2.73 − 2.52 (m, 3H), 2.16 − 2.10 (m, 1H), 1.23 − 1.07 (m, 12H). ^{13}C NMR (101 MHz, $CDCl_3$) δ 162.25 (s), 154.68 (s), 147.31 (s), 147.29 (s), 143.98 (d), 138.73 (s), 133.84 (s), 133.80 (s), 133.19 (d), 132.99 (s), 129.14 (d), 129.02 (d), 128.86 (d), 128.45 (d), 128.09 (d), 127.59 (d), 127.41 (d), 117.76 (s), 117.58 (s), 96.70 (d), 87.37 (d), 87.28 (d), 83.93 (d, J_{CP} = 2.9 Hz), 83.62 (d, J_{CP} = 6.6 Hz), 72.74 (d, J_{CP} = 16.1 Hz), 72.61 (d, J_{CP} = 16.8 Hz), 68.42 (t), 68.26 (t), 58.27 (t, J_{CP} = 19.0 Hz), 58.03 (t, J_{CP} = 19.0 Hz), 45.63 (d, J_{CP} = 4.4 Hz), 43.36 (d, J_{CP} = 12.4 Hz), 40.71 (t), 40.66 (t), (d), 24.64 (q), 24.58 (q), 24.53 (q), 24.50 (q), 20.38 (d, J_{CP} = 7.3 Hz), 20.35 (d, J_{CP} = 7.3 Hz). ^{31}P NMR (162 MHz, $CDCl_3$) δ 149.9, 149.1.
**11**	85%	Solvent system for flash chromatography: dichloromethane/acetone 4:1 + 1% triethylamine. TLC (dichloromethane/acetone 4:1): R_f = 0.19, 0.16. ^{1}H NMR (400 MHz, $CDCl_3$) δ 12.00 (s br., 1H), 9.19, 9.17 (2s br., 1H), 7.86 − 7.83 (m, 2H), 7.69 − 7.58 (m, 3H), 7.54 − 7.50 (m, 2H), 7.44 − 7.35 (m, 3H), 6.09 − 6.02 (m, 1H), 4.80 − 4.74, 4.70 − 4.65 (2m, 1H), 4.51 − 4.46, 4.42 − 4.38 (2m, 1H), 4.29 − 4.04 (m, 2H), 3.85 − 3.78 (m, 1H), 3.72 − 3.62 (m, 1H), 3.59 − 3.39 (m, 2H), 3.16 − 3.02 (m, 1H), 2.71 − 2.49, 2.39 − 2.33 (2m, 4H), 1.22 − 1.09 (m, 18H). ^{13}C NMR (101 MHz, $CDCl_3$) δ 179.33 (s), 179.19 (s), 155.60 (s), 155.53 (s), 147.86 (s), 147.79 (s), 147.51 (s), 147.49 (s), 147.34 (s), 147.21 (s), 138.76 (d), 138.62 (s), 138.40 (d), 138.57 (s), 133.45 (s), 133.35 (s), 132.82 (d), 132.60 (d), 129.20 (d), 128.9 (d), 128.96 (d), 128.36 (d), 128.34 (d), 128.05 (d), 127.90 (d), 127.35 (d), 127.31 (d), 122.90 (s), 122.62 (s), 117.92 (s), 117.72 (s), 86.21 (d), 85.89 (d), 83.64 (d), 83.58 (d), 74.16 (d, J_{CP} = 16.8 Hz), 73.51 (d, J_{CP} = 15.3 Hz), 69.40 (t), 68.97 (t), 57.93 (t, J_{CP} = 19.0 Hz), 57.68 (t, J_{CP} = 19.0 Hz), 45.31 (d, J_{CP} = 6.6 Hz), 43.32 (d, J_{CP} = 12.4 Hz), 38.28 (t), 38.23 (t), (d), 36.14 (d), 36.02 (d), 24.64 (q), 24.57 (q), 24.55 (q), 24.48 (q), 20.52 (d, J_{CP} = 7.3 Hz), 20.11 (d, J_{CP} = 6.6 Hz), 19.00 (q), 18.98 (q), 18.91 (q), 18.88 (q).
**12**	56%	Solvent system for flash chromatography: heptane/ethyl acetate 2:3 + 1% triethylamine. TLC (heptane/ethyl acetate 2:3): R_f = 0.23, 0.16. ^{1}H NMR (400 MHz, $CDCl_3$) δ 8.58 (s br., 1H), 7.92 − 7.88 (m, 2H), 7.72 − 7.69 (m, 2H), 7.55 − 7.52 (m, 2H), 7.45 − 7.35 (m, 3H), 7.33, 7.29 (2q, 1H, J = 1.3 Hz), 6.29 − 6.25 (m, 1H), 4.55 − 4.46 (m, 1H), 4.33 − 4.11 (m, 3H), 3.83 − 3.37 (m, 4H), 2.58 − 2.54 (m, 2H), 2.45 − 2.32 (m, 1H), 2.19 − 2.11 (m, 1H), 1.88, 1.87 (2d, 3H, J = 1.3 Hz), 1.24 − 1.06 (m, 12H). ^{13}C NMR (101 MHz, $CDCl_3$) δ 163.53 (s), 150.27 (s), 147.30 (s), 138.77 (s), 135.27 (d), 133.86 (s), 129.20 (d), 128.93 (d), 128.42 (d), 128.06 (d), 127.35 (d), 117.70 (s), 111.68 (s), 84.85 (d), 83.37 (d), 83.34 (d), 73.25 (d), 73.09 (d), 69.13 (t), 58.14 (t), 57.95 (t), 43.46 (d), 43.33 (d), 39.40 (t), 39.36 (t), 24.64 (q), 24.57 (q), 24.55 (q), 24.48 (q), 20.42 (t), 20.35 (t), 12.43 (q). ^{31}P NMR (162 MHz, $CDCl_3$) δ 149.6, 149.0.
